# Platinum-group metal half-sandwich complexes of 1-(α-d-glucopyranosyl)-4-hetaryl-1,2,3-triazoles: synthesis, solution equilibrium studies, and investigation of their anticancer and antimicrobial activities

**DOI:** 10.3389/fchem.2025.1619991

**Published:** 2025-09-01

**Authors:** Alshimaa I. Zaki, Adrienn Sipos, István Kacsir, Nóra Ildikó Kovács, Éva Kerekes, Evelin Szoták, Csongor Freytag, Máté Demény, István Révész, Péter Buglyó, Attila Bényei, Eszter Anna Janka, Gábor Kardos, László Somsák, Peter Bai, Éva Bokor

**Affiliations:** 1 Department of Organic Chemistry, University of Debrecen, Debrecen, Hungary; 2 Doctoral School of Chemistry, University of Debrecen, Debrecen, Hungary; 3 Department of Chemistry, Faculty of Science, Mansoura University, Mansoura, Egypt; 4 Department of Medical Chemistry, Faculty of Medicine, University of Debrecen, Debrecen, Hungary; 5 Department of Inorganic and Analytical Chemistry, Faculty of Sciences and Technology, University of Debrecen, Debrecen, Hungary; 6 One Health Institute, Faculty of Health Sciences, University of Debrecen, Debrecen, Hungary; 7 Department of Physical Chemistry, Faculty of Sciences and Technology, University of Debrecen, Debrecen, Hungary; 8 Department of Dermatology, Faculty of Medicine, University of Debrecen, Debrecen, Hungary; 9 Department of Metagenomics, University of Debrecen, Debrecen, Hungary; 10 National Public Health Centre, Budapest, Hungary; 11 NKFIH‐DE Lendület Laboratory of Cellular Metabolism, Debrecen, Hungary; 12 Research Center for Molecular Medicine, Faculty of Medicine, University of Debrecen, Debrecen, Hungary

**Keywords:** *N*-glycopyranosyl derivative, α-anomer, 1,2,3-triazole, half-sandwich complex, complex stability, cytostasis, bacteriostasis

## Abstract

Although platinum-based complexes are pivotal in chemotherapy, their clinical use is limited by toxicity and resistance. Previously, we identified a set of osmium, ruthenium, and iridium half-sandwich complexes of 1-*N*-(β-d-glucopyranosyl)-4-hetaryl-1,2,3-triazole-type N,N-chelators with potent and selective activity against a large set of diverse neoplasia cell models and multiresistant Gram-positive bacteria (methicillin-resistant *Staphylococcus aureus* (MRSA) and vancomycin-resistant *Enterococcus* (VRE)). Our aim in this study was to assess how the configuration of the C1 carbon in the glucose moiety affects the biological activity of the complexes. Thus, 1-*N*-(α-d-glucopyranosyl)-4-hetaryl-1,2,3-triazoles were synthesized and used as N,N-bidentate ligands to result in half-sandwich type complexes analogous to the earlier reported ones. Overall, the newly prepared complexes with the α-anomeric carbohydrate moiety had similar biological properties to the complexes with the β-anomeric carbohydrate unit in terms of their biological activity on cancer cells or primary human cells. Importantly, the bacteriostatic property of the complexes with an α-anomeric sugar moiety was inferior to that of the complexes containing the β-anomer.

## 1 Introduction

Platinum-based agents, as cisplatin, oxaliplatin, and carboplatin, represent pillars of chemotherapy regimens against numerous solid tumors and hematological malignancies (Kenny and Marmion, 2019; Yu et al., 2020; Zhang et al., 2022). Despite the versatility of the platins, their applicability is limited by platinum resistance and toxicity (Hartmann and Lipp, 2003; Mukherjea et al., 2020; McMullen et al., 2021; Sipos et al., 2021). This gap in the suitability of platins calls for the development of new organometallics (Boros et al., 2020; Bortolamiol et al., 2023; Singh et al., 2023; Omer et al., 2024) for which, among others, the complexes of other platinum-group metals (ruthenium (Melchart and Sadler, 2006; Hartinger et al., 2011; Zeng et al., 2017; Gichumbi and Friedrich, 2018; Meier-Menches et al., 2018; Coverdale et al., 2019; Kenny and Marmion, 2019; Štarha and Trávníček, 2019; Bashir et al., 2023; Prathima et al., 2023; Herić et al., 2024; Koziel et al., 2025), osmium (Hartinger et al., 2011; Hanif et al., 2014; Gichumbi and Friedrich, 2018; Konkankit et al., 2018; Meier-Menches et al., 2018; Štarha and Trávníček, 2019; Nabiyeva et al., 2020; Li et al., 2021; Prathima et al., 2023), iridium (Leung et al., 2013; Liu and Sadler, 2014; Gichumbi and Friedrich, 2018; Konkankit et al., 2018; Štarha and Trávníček, 2019; Prathima et al., 2023; Koziel et al., 2025; Mansour et al., 2025; Štarha, 2025), or rhodium (Leung et al., 2013; Gichumbi and Friedrich, 2018; Štarha and Trávníček, 2019; Máliková et al., 2021; Prathima et al., 2023; Saha et al., 2025)) appear to be potential candidates due to better toxicity profiles compared to platinum-based drugs (Mello-Andrade et al., 2018; Gano et al., 2019; Liu et al., 2019; Mihajlovic et al., 2020). In line with that, three ruthenium derivatives, NAMI-A (Leijen et al., 2015), KP1019/1339 (IT-139, BOLD100) (Burris et al., 2016), and TLD-1433 (Kulkarni et al., 2022) are already in various phases of clinical trials against neoplastic diseases such as bladder or lung cancer.

A subgroup of bioactive platinum-group metal complexes is the half-sandwich complexes with anticancer (Melchart and Sadler, 2006; Liu and Sadler, 2014; Gichumbi and Friedrich, 2018; Nabiyeva et al., 2020; Máliková et al., 2021; Bashir et al., 2023; Koziel et al., 2025; Mansour et al., 2025; Štarha, 2025) or even with antibacterial (Karpin et al., 2013; DuChane et al., 2018; Kljun et al., 2018; Lapasam et al., 2020a; Lapasam et al., 2020b; Lapasam et al., 2020c; Yufanyi et al., 2020; Bernier et al., 2021; Coverdale et al., 2021; Klaimanee et al., 2021; Balázs et al., 2022; Dimitrijevic et al., 2023; Kacsir et al., 2023a; Kacsir et al., 2023b), antiparasitic (Martínez et al., 2012; Desiatkina et al., 2020; Mbaba et al., 2020; Milheiro et al., 2020; Fandzloch et al., 2021; Holzer et al., 2022; Gambino, 2024), antiviral (Yufanyi et al., 2020; Chuong et al., 2021; Jankovic et al., 2022), and antifungal (Kljun et al., 2014; Klaimanee et al., 2021; Dimitrijevic et al., 2023) properties. We have recently synthesized a series of half-sandwich complexes with β-d-glycopyranosyl azole-type N,N-bidentate ligands and revealed their anticancer and antibacterial potentials (Kacsir et al., 2021; Balázs et al., 2022; Kacsir et al., 2022; Kacsir et al., 2023a).

**FIGURE 1:**
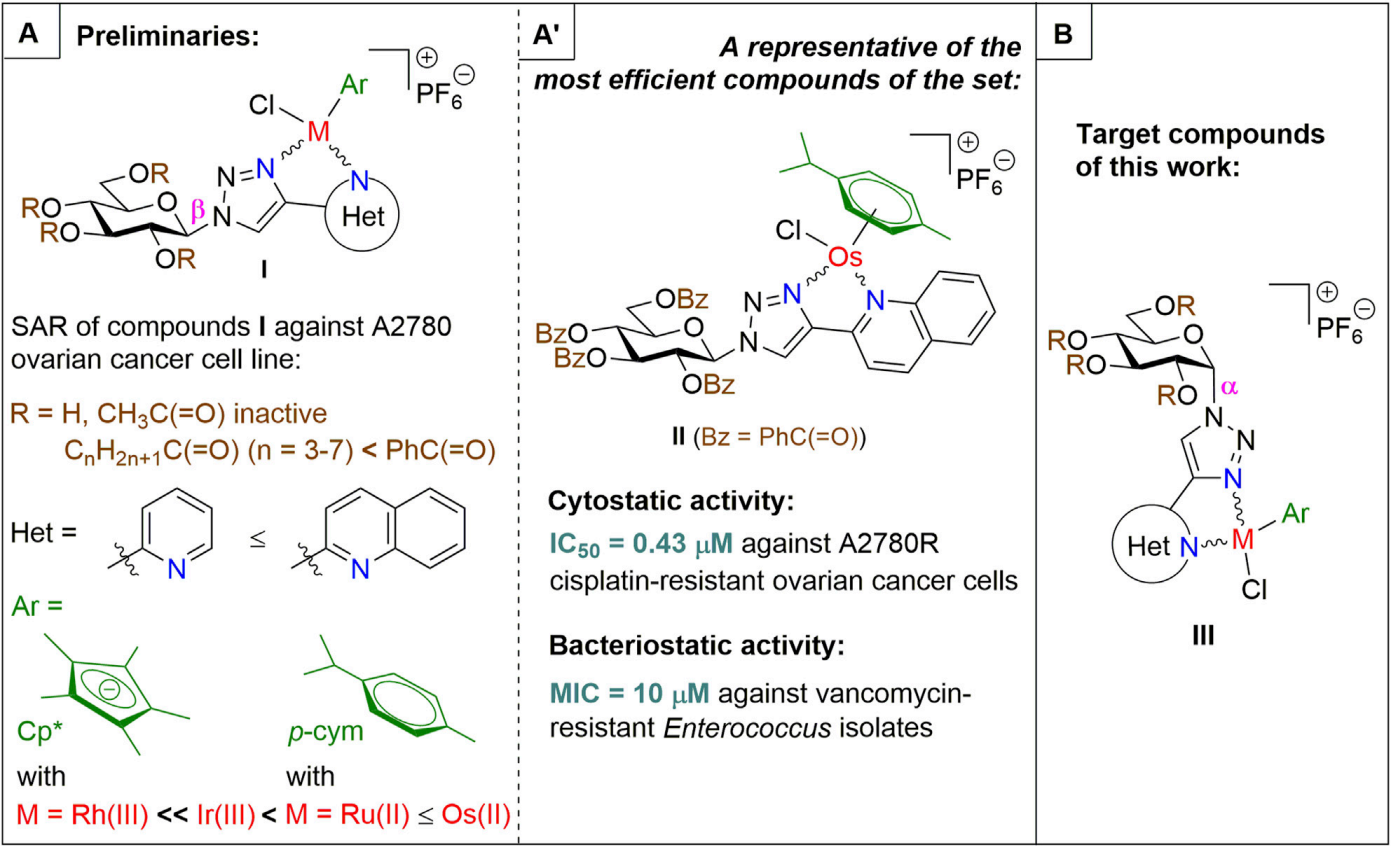
Outline of the SAR study for our previously published glucose-derived half-sandwich type complexes **(A)**, a biologically active representative of the set **(A′)**, and target compounds of the present work **(B)**.

Among them, the complexes of 1-*N*-(β-d-glucopyranosyl)-4-hetaryl-1,2,3-triazoles (Figure 1A, **I**) proved to be the most promising subset with members (e.g., **II** in Figure 1A′) displaying cytostatic properties with submicromolar IC_50_ values against a plethora of cellular models of various neoplasia (carcinomas, sarcomas and hematological malignancies), and also showing bacteriostatic activity with low micromolar MIC values on multiresistant Gram-positive bacteria (vancomycin-resistant *Enterococcus* (VRE) and methicillin-resistant *Staphylococcus* *aureus* (MRSA)). In addition, these complexes, by exerting at least one order of magnitude lower IC_50_ on cancer or bacterial cells than those values on human primary dermal fibroblasts, had definite selectivity (Kacsir et al., 2021; Balázs et al., 2022; Kacsir et al., 2022; Kacsir et al., 2023a). The complexes elicited oxidative stress in mammalian cells (Kacsir et al., 2021; Kacsir et al., 2022; Kacsir et al., 2023a) and in bacteria (Kacsir et al., 2023a), which could be a key process underlying their biological activity.

The main findings regarding the structure–activity relationships (SAR) of compounds **I** are outlined in Figure 1A (for details, the reader is kindly asked to survey our previous publications (Kacsir et al., 2021; Balázs et al., 2022; Kacsir et al., 2022; Kacsir et al., 2023a)). The most important feature regarding biological activity is the presence of *O*-protected (preferably *O*-perbenzoylated) carbohydrate moieties, which confer lipophilicity and likely facilitate cooperative target binding, while the removal of the protecting groups abolishes the biological activity of the complexes (Kacsir et al., 2021; Balázs et al., 2022; Kacsir et al., 2022; Kacsir et al., 2023a).

In this work, we will extend the SAR of **I** by an additional modification in the sugar part of the N,N-bidentate ligands (Figure 1B), namely, the application of 1-*N*-(α-d-glucopyranosyl)-4-hetaryl-1,2,3-triazoles (**III**) as ligands. The change of the anomeric configuration from β to α alters the orientation of the heterocyclic aglycon part of the glucose-derived N,N-chelators and that of the coordination sphere of the complexes. Thus, the main goal of this study is to assess how this modification in the molecular shape/geometry affects the biological effectiveness of this type of half-sandwich complex.

## 2 Results

### 2.1 Syntheses

The synthetic work was started with the preparation of the glucose-derived N,N-bidentate ligands (Table 1). First, Cu(I)-catalyzed cycloaddition of *O*-peracetylated α-d-glucopyranosyl azide (Zhang et al., 1999) **1** with 2-ethynylpyridine and -quinoline was carried out under standard CuAAC conditions (Wilkinson et al., 2006; Kraft et al., 2015), using a CuSO_4_/l-ascorbic acid catalyst system in aqueous *tert*-butyl alcohol to get *O*-peracetylated 1-(α-d-glucopyranosyl)-4-hetaryl-1,2,3-triazoles (**2a**,**b**) in moderate to high yields. Compounds **2a,b** were subsequently subjected to Zemplén deacetylation to afford the unprotected derivatives **3a,b** in excellent yields. Treatment of **3a,b** with benzoyl chloride in pyridine resulted in the corresponding *O*-perbenzoylated analogs **4a,b** in high yields. Additionally, the hydroxyl groups of compound **3a** were esterified by pentanoyl chloride to obtain ligand **5a** in acceptable yield.

**TABLE 1:** Synthesis of 1-(α-d-glucopyranosyl)-4-hetaryl-1,2,3-triazoles.

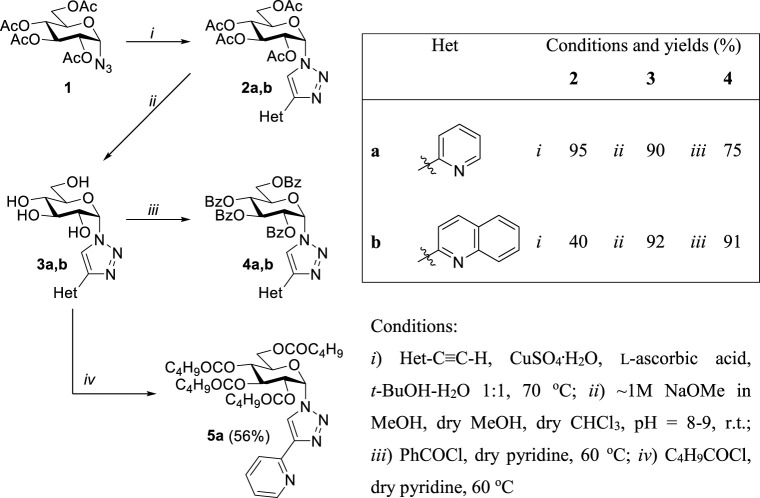

With the new glucose-based heterocyclic ligands in our hands, the synthesis of the target complexes, by the adaptation of our earlier published method (Kacsir et al., 2021; Kacsir et al., 2022; Kacsir et al., 2023a), was the next step (Table 2 and Table 3).

**TABLE 2:** Synthesis of half-sandwich (η^6^-*p*-cym)Ru(II) and -Os(II) complexes with 1-(α-d-glucopyranosyl)-4-hetaryl-1,2,3-triazoles.

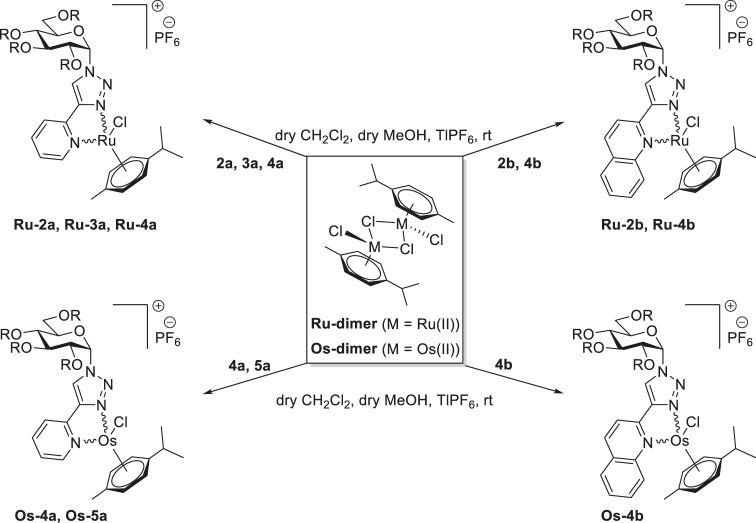					
Entry	Ligand	R	Product	Yield (%)	Diastereomeric ratio
1	**2a**	Ac	**Ru-2a**	81	6:5
2	**2b**	Ac	**Ru-2b**	96	7:1
3	**3a**	H	**Ru-3a**	44	1:1
4	**4a**	Bz	**Ru-4a**	91	2:1
5	**4a**	Bz	**Os-4a**	87	4:1
6	**4b**	Bz	**Ru-4b**	98	5:1
7	**4b**	Bz	**Os-4b**	96	5:2
8	**5a**	C_4_H_9_C(=O)	**Os-5a**	90	1:1

**TABLE 3:** Synthesis of half-sandwich (η^5^-Cp*)Ir(III) and -Rh(III) complexes with 1-(α-d-glucopyranosyl)-4-hetaryl-1,2,3-triazoles.

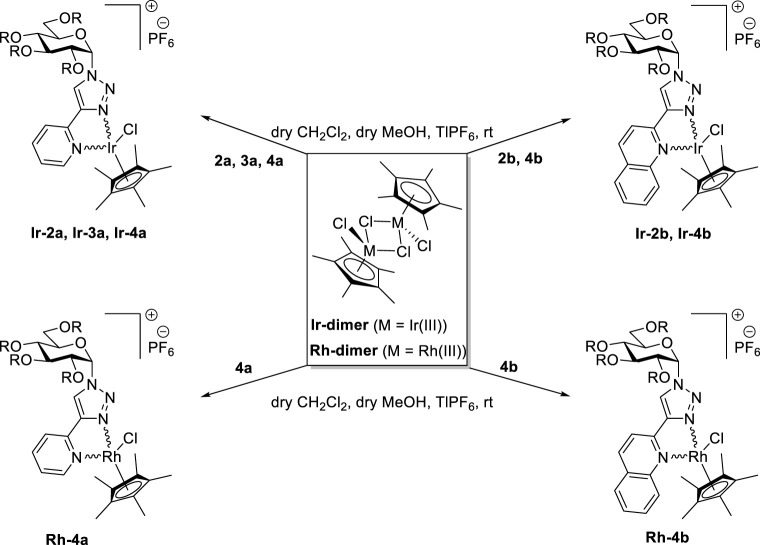					
Entry	Ligand	R	Product	Yield (%)	Diastereomeric ratio
1	**2a**	Ac	**Ir-2a**	87	5:1
2	**2b**	Ac	**Ir-2b**	87	4:1
3	**3a**	H	**Ir-3a**	51	1:1
4	**4a**	Bz	**Ir-4a**	91	3:2
5	**4a**	Bz	**Rh-4a**	91	1:1
6	**4b**	Bz	**Ir-4b**	97	3:2
7	**4b**	Bz	**Rh-4b**	70	3:2

Accordingly, the 2-pyridyl substituted 1,2,3-triazoles **2a‒5a** were reacted with dichloro (η^6^-*p*-cymene)Ru(II) and -Os(II) dimers (**Ru-dimer** and **Os-dimer**) in the presence of TlPF_6_ in a mixture of CH_2_Cl_2_ and MeOH (Table 2) to give the desired cationic half-sandwich type Ru(II) and Os(II) complexes **Ru-2a‒Ru-4a** (entries 1,3,4), **Os-4a** (entry 5), and **Os-5a** (entry 8) with a PF_6_^−^ counterion. Treatment of the 2-quinolyl derivatives **2b** and **4b** with the same chloro-bridged dimers was also performed (Table 2), resulting in further *p*-cymene-containing complexes **Ru-2b** (entry 2), **Ru-4b** (entry 6), and **Os-4b** (entry 7).

The complexation of the above ligands was also accomplished with dichloro (η^5^-pentamethylcyclopentadienyl)Ir(III) and Rh(III) dimers (**Ir-dimer** and **Rh-dimer**, Table 3) furnishing half-sandwich- type (η^5^-Cp*)Ir(III) complexes **Ir-2a,b** (entries 1 and 2), **Ir-3a** (entry 3), **Ir-4a,b** (entries 4 and 6), and Rh(III)-based analogs **Rh-4a,b** (entries 5 and 7).

Similar to previously reported complexes with 1-(β-d-glucopyranosyl)-4-hetaryl-1,2,3-triazoles (Kacsir et al., 2021; Kacsir et al., 2022; Kacsir et al., 2023a), all of the newly prepared complexes presented in Tables 2 and 3 were obtained as mixtures of two diastereoisomers.

### 2.2 X-ray structure of complex Ir-3a

**FIGURE 2:**
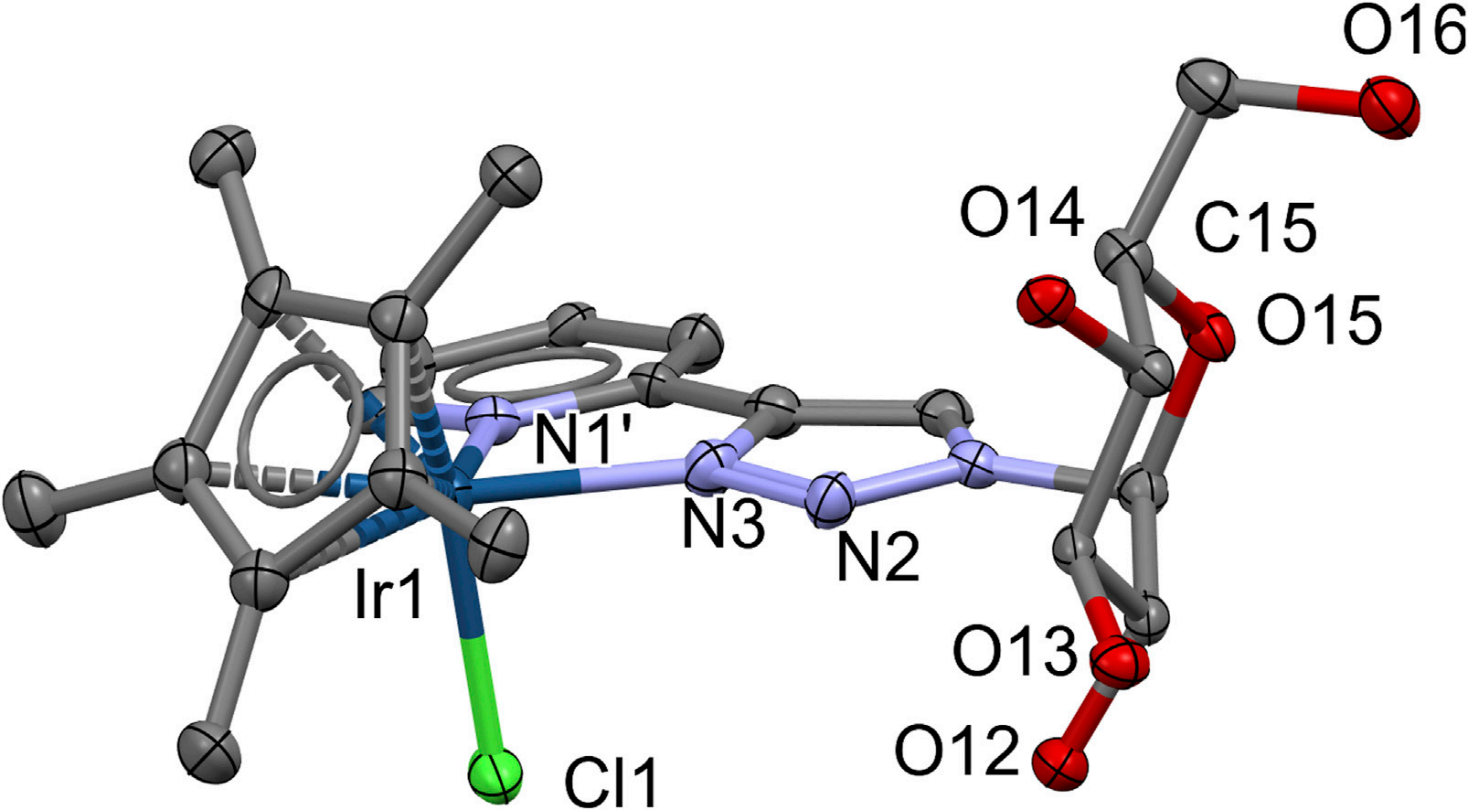
View of **Ir-3a** with a partial numbering scheme. Hydrogen atoms, solvent molecules, and counter ion PF_6_^−^ are omitted for clarity.

A single crystal of one of the isomers of **Ir-3a** could be obtained by slow evaporation of the solution of the corresponding complex in *i*-propyl alcohol at room temperature. The X-ray crystallography analysis of this sample confirmed the presumed coordination mode with the 5-membered chelate ring formed by the assistance of the glucose-based heterocyclic N,N-bidentate ligand **3a** (Figure 2). The asymmetric unit, apart from the PF_6_^−^ counterion, contains *i*-propyl alcohol and two water molecules. The coordination of the complex is the expected one. For example, the angle between the plane of the Cp* ring and the (Ir,N,N) plane is 57 degrees, which is in good agreement with other similar structures found in the Cambridge Structural Database. Moreover, the sugar moiety and the pyridinyl-triazole ring system make an L-shape, as can be seen in Figure 2. CCDC deposition number: 2440135 for **Ir-3a** contains the supplementary crystallographic data for this article.^1^

### 2.3 Interaction of ligands 3a and 3b with [(η^6^-*p*-cym)Ru]^2+^ and [(η^5^-Cp*)Rh]^2+^ cations in solution

**FIGURE 3:**
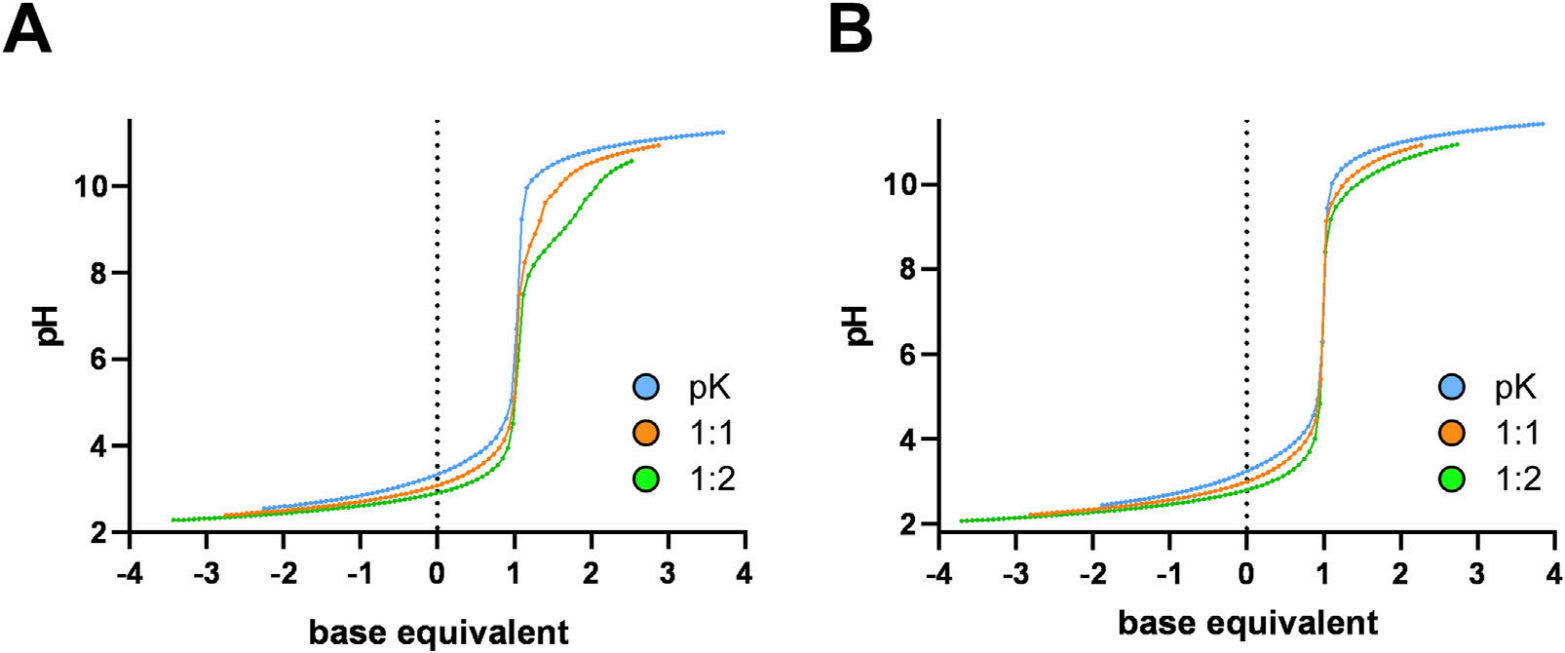
Representative titration curves registered in the H^+^–ligand („pK”) and in the [(η^6^-*p*-cym)Ru]^2+^-**3a** ligand system at various metal ion to ligand ratios **(A)**; Representative titration curves registered in the H^+^–ligand (pK”) and in the [(η^5^-Cp*)Rh]^2+^-**3a** ligand system at various metal ion to ligand ratios **(B)**. Negative base equivalent refers to an excess of acid in the sample.

In order to obtain information on the acidbase character of the ligands and on the stoichiometry and stability of the complexes present in aqueous medium, potentiometric titrations were carried out in selected systems. Based on the results, both **3a** and **3b**, having good enough solubility in water, are capable of releasing one hydrogen ion from the fully protonated forms in the measurable pH range (see Figure 3, “pK”). The calculated p*K*_a_ values appear in Table 4; these values belong to the N-heterocyclic ring of the ligands. The slightly lower values compared to those of the parent N-donors (pyridine: 5.25 (Chmurzyński, 2000), quinoline: 4.93 (Wu et al., 1995)) can be interpreted by the close vicinity of the triazole rings making the pyridine or quinoline N-s less basic in these novel ligands, even though an internal hydrogen bond formation is possible. For comparison, the previously prepared β-anomeric pair of **3a**, compound **3a-β** (Kacsir et al., 2021) in Table 4, was also studied. Notably, there is no significant difference in the acid-base character of the two anomers (Table 4).

**TABLE 4:** Protonation constants (p*K*_a_) of the ligands studied and stability constants (log*β*) of the ML complexes with the [(η^5^-Cp*)Rh]^2+^ metal ion.

Ligand	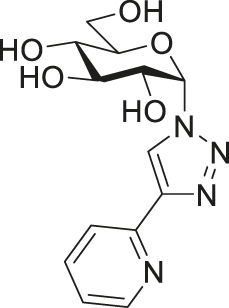	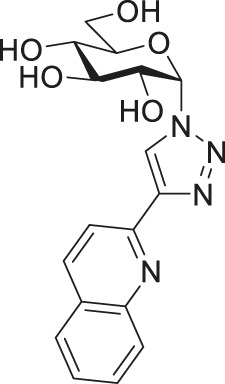	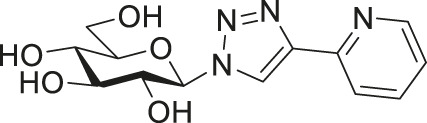
	**3a**	**3b**	**3a**-**β** (Kacsir et al., 2021)
**p*K***_***a***_	3.59 (1)	3.56 (1)	3.44 (2)
	**[(η** ^**5**^ -Cp*)Rh] ^**2+**^		
**log*β***_**[ML]**_	5.50 (4)	5.55 (3)	5.12 (2)

Because the studied organoosmium and -iridium cations are known to form complexes in slow processes, they were not suitable for direct pH-potentiometric titrations. Complexes containing the [(η^6^-*p*-cym)Ru]^2+^ entity were found to be active in previous biological studies (Kacsir et al., 2021; Kacsir et al., 2022; Kacsir et al., 2023a; Kacsir et al., 2023b). Therefore, solution equilibrium studies were first performed in the presence of this metal ion. Representative titration curves are shown in Figure 3A. With this metal ion, reaching pH equilibrium was also found to be a very slow process; under the measuring conditions, no steady data points could be obtained. At the same time, the tentative curves indicate a moderate pH effect of the metal ion that can be seen in the acidic pH range due to the low basicity of the ligands. Above pH ∼ 7.0, however, extra base consumption processes occur, presumably due to partial hydrolysis of the metal ion. Due to uncertain data points, the titration curves registered in the organoruthenium samples could not be evaluated.

Although its complexes did not exhibit considerable activity either, to model the solution equilibrium processes, the organorhodium ion was also involved in these studies. In this case, a smaller pH effect was observed in the acidic pH range (Figure 3B); however, unlike the organoruthenium system, no hydrolysis of the metal ion is noticeable in the range 5.5 < pH < 9.0, revealing the presence of complexed species only. Evaluation of the titration curves in the range 2.0 < pH < 7.0 resulted in very simple models with a single complex, [ML]^2+^, in which the (N,N) coordination of the ligands is assumed. Comparing the two metal ion containing systems, the complexation with [(η^6^-*p*-cym)Ru]^2+^, starts at a more acidic pH, but hydrolytic processes occur already in the physiological pH range due to the higher affinity of the metal ion to hydrolysis. For the [(η^5^-Cp*)Rh]^2+^, these processes are shifted towards higher pH; therefore, the [RhL]^2+^ species is still present at physiological pH. The rather similar values of the [RhL] complexes (Table 4) for the three ligands may indicate that the presence of the second aromatic ring in **3b** and the orientation of the triazole ring for pyridine-containing ligands (**3a** and **3a-β**) have no significant effect on the stability of the chelate formed with the metal ion.

### 2.4 Biological characterization of the complexes

#### 2.4.1 The complexes do not exert rapid toxicity but impair mitochondrial respiration

**FIGURE 4:**
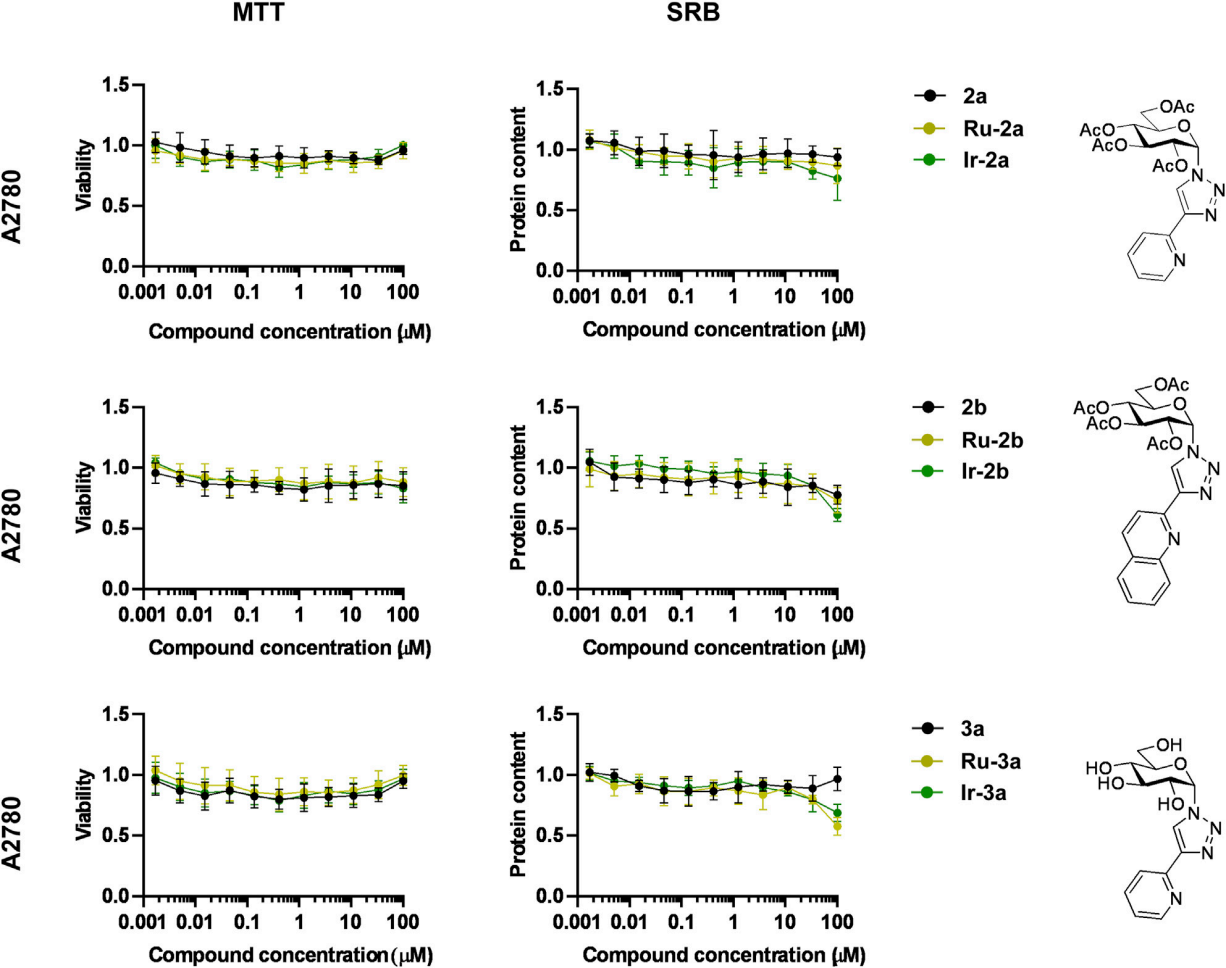
The ruthenium and iridium complexes of ligands **2a**, **2b**, and **3a** do not influence cell viability and cell proliferation. For MTT assays, 4 × 10^3^ A2780 cells were plated into 96-well plates, and for SRB assays, 1.5 × 10^3^ A2780 cells were plated into 96-well plates. Cells were treated with the compounds in the concentrations indicated for either 4 h for an MTT assay or for 48 h for an SRB assay. Data are represented as average ± SD, from four biological replicates, with the exception of the MTT dataset of **Ir-2b** and the SRB datasets of **2b**, **Ru/Ir-2b,** and **Ir-3a**, which were derived from three biological replicates. Individual assays were performed in duplicate. Values were normalized for vehicle-treated cells, and the absorbance for vehicle-treated cells equals 1. Data are represented as fold change compared to vehicle-treated controls. Normality was assessed using the D’Agostino–Pearson test. Statistical significance was assessed using a two-way ANOVA test followed by Tukey’s post hoc test. For better visibility, normality, transformations, statistical tests, and p values are presented in an MS Excel sheet at https://figshare.com/s/3a0aa60b66f3f746f41e. Nonlinear regression was performed on the datasets; IC_50_ values are indicated in Table 5. Color code: black—free ligand (**2a,b** or **3a**), khaki—ruthenium complex (**Ru-2a,b** or **Ru-3a**), green—iridium complex (**Ir-2a,b** or **Ir-3a**).

**FIGURE 5:**
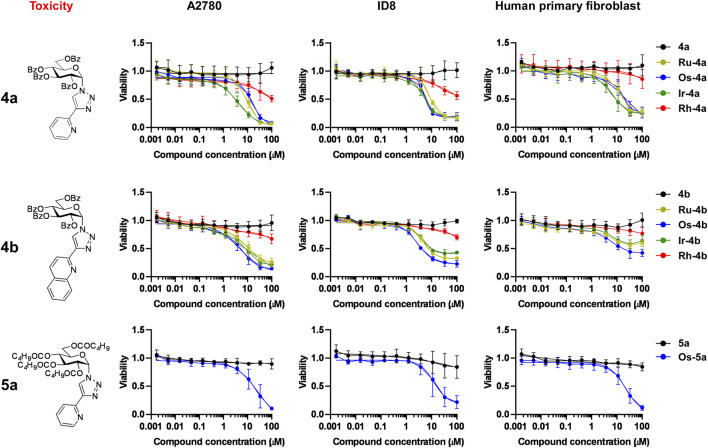
The ruthenium, osmium, and iridium complexes of ligands **4a**, **4b**, and **5a** decrease MTT reduction. For MTT assays, 4 × 10^3^ A2780 cells, 3 × 10^3^ ID8 cells, or 6 × 10^3^ human primary fibroblasts were plated into 96-well plates. Cells were treated with the compounds in the concentrations indicated for 4 h. Data are represented as average ± SD, from four biological replicates on A2780, and from three biological replicates on ID8 and human primary fibroblasts. For the datasets of **5a** and **Os-5a** on human primary fibroblasts, the data represent four biological replicates. Individual assays were performed in duplicate. Values were normalized for vehicle-treated cells, and the absorbance for vehicle-treated cells equals 1. Normality was assessed using the D’Agostino–Pearson test. Statistical significance was assessed using a two-way ANOVA test followed by Tukey’s post hoc test. For better visibility, normality, transformations, statistical tests, and p values are presented in an MS Excel sheet at https://figshare.com/s/3a0aa60b66f3f746f41e. Nonlinear regression was performed on the datasets; IC_50_ values are indicated in Table 5. Color code: black—free ligand (**4a,b** or **5a**), khaki—ruthenium complex (**Ru-4a,b**), green—iridium complex (**Ir-4a,b**), blue—osmium complex (**Os-4a,b** or **Os-5a**), and red—rhodium complex (**Rh-4a,b**).

The first step in the biological characterization of the complexes was the screening for the capacity of the complexes to exert rapid toxicity, that is, cell death, using the MTT assay. None of the ligands (**2a**, **2b**, **3a**, **4a**, **4b**, **5a**) induced toxicity in ovarian cancer cells (A2780 and ID8 cells) or human primary dermal fibroblasts (Figures 4, 5; Table 5). Complexes in which the hydroxyl groups of the carbohydrate moiety were unprotected or esterified by acetyl groups (**Ru/Ir-2a,b**, **Ru/Ir-3a**) did not induce cell death in A2780 cells in accordance with our previous observations (Kacsir et al., 2021; Kacsir et al., 2022; Kacsir et al., 2023a; Kacsir et al., 2023b) (Figure 4; Table 5). However, complexes of the *O*-perbenzoylated ligands **4a** and **4b** or the *O*-perpentanoylated ligand **5a**, to our surprise, induced MTT reduction that is a sign of acute toxicity in ovarian cancer cells (A2780 and ID8 cells), as well as in the untransformed human primary dermal fibroblasts (Figure 5; Table 5). This finding contrasted with our previous observations as the complexes with the β-configured glucose moiety did not induce toxicity (Kacsir et al., 2021; Kacsir et al., 2022; Kacsir et al., 2023a). Namely, the ruthenium, osmium, and iridium complexes of ligands **4a**, **4b**, and **5a** (**Ru/Os/Ir-4a,b**, **Os-5a**) induced acute toxicity, while the rhodium complexes of ligands **4a** and **4b** (**Rh-4a**, **Rh-4b**) did not have such property on either of the cell lines assessed (Figure 5; Table 5).

**TABLE 5:** The distribution coefficients (logD), the maximal inhibitory, IC_50_, and Hill coefficient values of the complexes. n.c. – cannot be calculated.

	logD	A2780						ID8						Fibroblast					
		MTT			SRB			MTT			SRB			MTT			SRB		
		max %	IC_50_ (μM)	Hill	max %	IC_50_ (μM)	Hill	max %	IC_50_ (μM)	Hill	max %	IC_50_ (μM)	Hill	max %	IC_50_ (μM)	Hill	max %	IC_50_ (μM)	Hill
**2a**		0			0														
**Ru-2a**	−2.13	0			0														
**Ir-2a**	−1.77	0			0														
**2b**		0			22.33														
**Ru-2b**	−1.15	0			26.98														
**Ir-2b**	−0.91	0			38.84														
**3a**		0			0														
**Ru-3a**	−1.96	0			42.35														
**Ir-3a**	−2.53	0			31.25														
**4a**		0			29.51			0			0			0			11.89		
**Ru-4a**	+1.88	>90	8.83	1.51	>90	1	1.98	82.06	8.12	2.12	>90	3.73	2.17	71.00	12.67	1.36	89.13	6.36	2.07
**Os-4a**	+1.67	>90	14.42	1.57	>90	0.85	1.91	80.66	5.01	2.77	>90	3.11	n.c.	73.90	24.18	0.79	87.90	35.64	0.91
**Ir-4a**	+1.87	>90	3.83	1.06	>90	0.73	1.73	82.08	4.99	1.71	>90	2.13	2.73	72.70	5.81	1.23	82.44	7.30	1.71
**Rh-4a**	+1.79	48.92			>90	n.c	n.c.	43.68			0			14.61			0		
**4b**		0			0			0			0			0			10.76		
**Ru-4b**	+1.61	73.80	9.00	0.78	>90	1.28	1.84	66.85	4.31	1.73	>90	1.38	2.30	43.85			64.73	12.40	2.61
**Os-4b**	+1.39	85.32	4.77	0.85	>90	0.65	1.94	77.24	2.80	1.37	>90	0.93	3.51	57.66			84.53	10.54	2.71
**Ir-4b**	+1.89	78.26	17.61	0.43	>90	1.18	1.71	58.48	3.50	1.46	>90	n.c.	n.c.	42.41			47.88		
**Rh-4b**	+1.64	32.64			75.23			30.06			0			23.08			0		
**5a**		0			33.92			15.89			0			15.72			27.97		
**Os-5a**	+1.97	>90	28.85	0.88	>90	2.17	1.86	78.16	12.39	1.55	>90	4.92	3.44	88.29	22.4	1.35	>90	21.76	2.47

	Capan2			U2OS			L428			Cisplatin-resistant A2780					
	SRB			SRB			SRB			MTT			SRB		
	max %	IC_50_ (μM)	Hill	max %	IC_50_ (μM)	Hill	max %	IC_50_ (μM)	Hill	max %	IC_50_ (μM)	Hill	max %	IC_50_ (μM)	Hill
**2a**															
**Ru-2a**															
**Ir-2a**															
**2b**															
**Ru-2b**															
**Ir-2b**															
**3a**															
**Ru-3a**															
**Ir-3a**															
**4a**															
**Ru-4a**	>90	2.60	3.30	>90	9.07	2.95	88.72	17.16	2.50	74.47	11.43	1.46	>90	2.70	2.66
**Os-4a**	>90	2.09	1.64	>90	10.8	3.08	>90	19.76	3.56	48.49	n.c.	n.c.	>90	1.99	2.40
**Ir-4a**	>90	1.99	2.35	>90	6.58	2.99	87.35	n.c.	n.c.	75.69	8.08	1.97	>90	2.43	3.43
**Rh-4a**	47.71			0			52.46			31.38			74.37	n.c.	n.c.
**4b**															
**Ru-4b**	>90	2.20	2.91	88.19	4.26	3.07	>90	2.64	2.42	19.79			>90	3.57	2.63
**Os-4b**	>90	0.96	2.24	>90	3.65	2.66	>90	n.c.	n.c.	40.32			>90	2.56	2.43
**Ir-4b**	>90	1.45	2.15	>90	4.98	1.98	>90	n.c.	n.c.	25.35			>90	2.88	2.13
**Rh-4b**	61.73			0			>90	6.36	0.92	0			30.30		
**5a**															
**Os-5a**	>90	5.25	2.85	>90	20.40	2.05	>90	5.10	3.40	68.17			86.36	3.63	3.30

**FIGURE 6:**
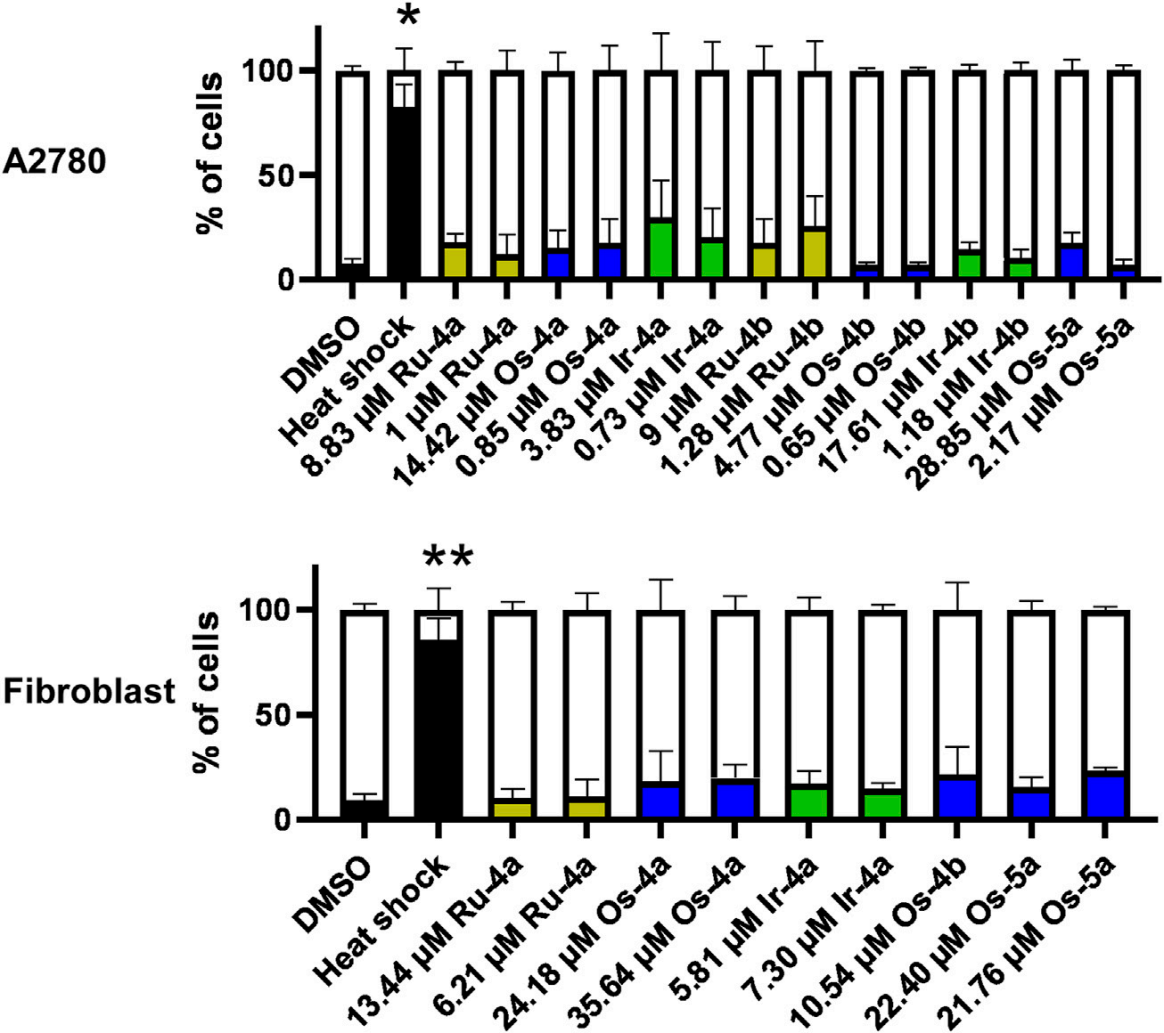
The ruthenium, osmium, and iridium complexes of ligands **4a**, **4b**, and **5a** do not induce cell death. For the assay, 2 × 10^6^ A2780 cells or 8 × 10^5^ fibroblasts were plated into 12-well plates and were treated with the indicated complexes at the indicated concentrations for 4 h. Cells undergoing heat shock were used as the positive control. Cells were then stained with annexin V and propidium iodide (PI) and subjected to flow cytometry as described in *Materials and Methods*. The percentage of cells in the double negative quadrant (in the white part of the bar) and the sum of the remaining three quadrants (i.e., dead cells; filled part of the bar) are depicted. Data are represented as average ± SD, from three biological replicates. Statistical analysis was performed on the proportion of dead cells in each sample set, comparing treatments to the vehicle (DMSO) control. Normality was assessed using the Shapiro–Wilk’s test, and the Kruskal–Wallis test was applied, followed by Dunn’s post hoc test. * and ** symbolize statistically significant differences between the indicated cohort and the DMSO-treated cells at p < 0.05 or 0.01, respectively. Color code: black—DMSO-treated or heat-shocked cells, khaki—ruthenium complex (**Ru-4a,b**), green—iridium complex (**Ir-4a,b**), and blue—osmium complex (**Os-4a,b** or **Os-5a**).

To verify the toxicity-inducing behavior of the complexes with β (non-toxic) and α (slightly toxic) anomeric sugar moiety, we complemented the MTT assay with the propidium iodide–annexin V-FITC double staining in case of the complexes that efficiently reduced the MTT signal in A2780 or primary fibroblast cells (complexes of ligands **4a**, **4b**, and **5a**). All compounds were applied in concentrations corresponding to the IC_50_ value obtained in MTT assays and in SRB assays (SRB assays are discussed later) on A2780 cells and primary human dermal fibroblasts. Assessing these concentrations ensured that we could detect acute toxicity in concentrations that induced long-term cytostasis. From our perspective, it was not important to distinguish between apoptosis and necrosis. We summed up propidium iodide, annexin V, and double-positive cells as dead cells and contrasted them with double-negative (living) cells. Importantly, the active complexes of **4a**, **4b**, and **5a** induced only marginal cell death (Figure 6) that was comparable to previous observations with the complexes of ligands incorporating a β-d-glucopyranosyl moiety (Kacsir et al., 2021; Kacsir et al., 2022; Kacsir et al., 2023a; Kacsir et al., 2023b).

#### 2.4.2 Complexes with benzoyl- and pentanoyl-protected glucose units have selective cytostatic properties

**FIGURE 7:**
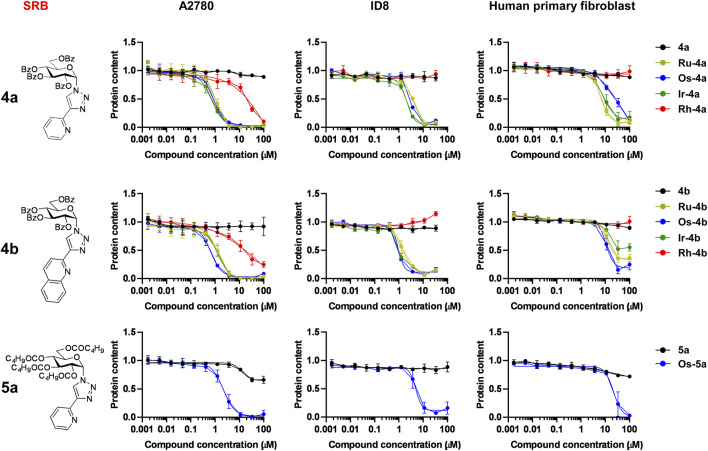
The ruthenium, osmium, and iridium complexes of ligands **4a**, **4b**, and **5a** are cytostatic on ovarian cancer models with a good therapeutic index. For SRB assays, 1.5 × 10^3^ A2780 cells, 1.5 × 10^3^ ID8 cells, or 4 × 10^3^ primary fibroblasts were plated into 96-well plates. Cells were treated with the compounds in the concentrations indicated for 48 h. Data are represented as average ± SD, from four biological replicates on A2780 and human primary fibroblasts, except in the case of Os complexes, where experiments were performed in three biological replicates. ID8 cells were assessed in three biological replicates. Individual assays were performed in duplicate. Values were normalized for vehicle-treated cells, and the absorbance for vehicle-treated cells equals 1. Normality was assessed using the D’Agostino–Pearson test. Statistical significance was assessed using a two-way ANOVA test followed by Tukey’s post hoc test. For better visibility, normality, transformations, statistical tests, and p values are presented in an MS Excel sheet at https://figshare.com/s/3a0aa60b66f3f746f41e. Nonlinear regression was performed on the datasets; IC_50_ values are indicated in Table 5. Color code: black—free ligand (**4a,b** or **5a**), khaki—ruthenium complex (**Ru-4a,b**), blue—osmium complex (**Os-4a,b** or **Os-5a**), green—iridium complex (**Ir-4a,b**), and red—rhodium complex (**Rh-4a,b**).

We assessed the cytostatic properties of the complexes using the sulforhodamine B (SRB) assay (Skehan et al., 1990). None of the free ligands (**2a,b**, **3a**, **4a,b**, **5a**) proved to be cytostatic on any of the cell lines used in the study (Figures 4, 7; Table 5). Similar to the toxicity results, complexes in which the OH-groups of the glucose moiety were not protected (**Ru/Ir**-**3a**) or were *O*-peracetylated (**Ru/Ir**-**2a**,**b**) did not exert cytostasis (Figure 4; Table 5). In contrast to that, complexes containing *O*-perbenzoylated (**Ru/Os/Ir-4a**,**b**) or -pentanoylated (**Os-5a**) sugar units exerted cytostasis in all cell lines (Figure 7). Of the *p*-cym-Ru(II), *p*-cym-Os(II), and Cp*-Ir(III) and Cp*-Rh(III) complexes of **4a** and **4b** (**Ru/Os/Ir/Rh-4a,b**), the latter ones (**Rh-4a**,**b**) were markedly less efficient or were ineffective compared to their counterparts **Ru/Os/Ir-4a,b** in all model systems (Figure 7). The IC_50_ values of the *p*-cym-Ru(II), *p*-cym-Os(II), and Cp*-Ir(III) complexes of ligands **4a**, **4b**, and **5a** were in the low micromolar-to-submicromolar range in both A2780 and ID8 ovarian cancer cells (Figure 7; Table 5). The quinoline-containing complexes (complexes of **4b**) had better IC_50_ values than their pyridine-containing pairs (complexes of **4a** and **5a**) (Figure 7; Table 5). Importantly, the IC_50_ values of the same complexes on primary human dermal fibroblasts were one order of magnitude higher than their IC_50_ values on A2780 and ID8 ovarian cancer cells (Figure 7; Table 5), suggesting a reasonable therapeutic index.

#### 2.4.3 The bioactive complexes have cytostatic properties on a larger set of neoplasias, similar to their cytostatic properties on primary human fibroblasts

**FIGURE 8:**
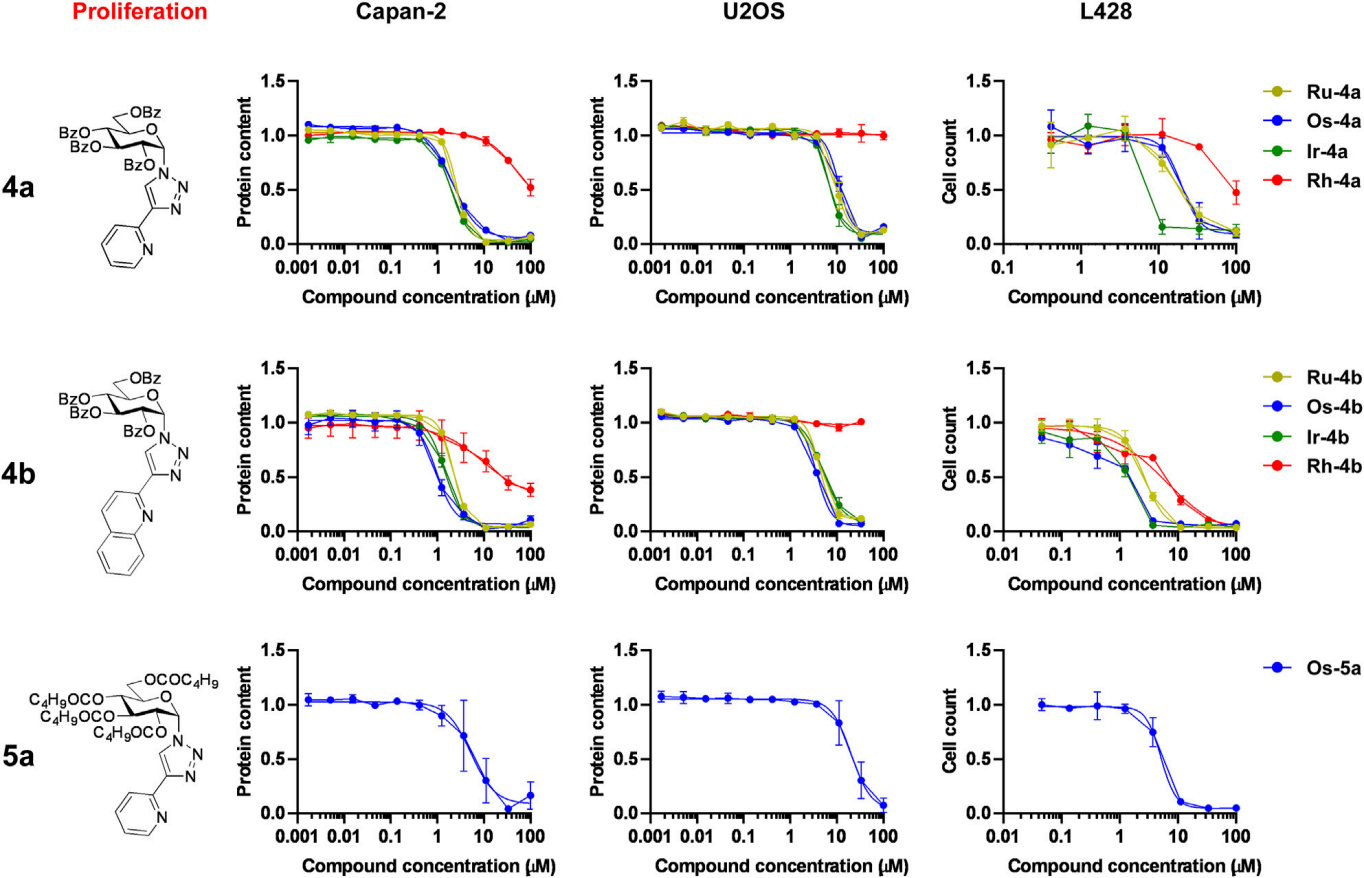
The complexes of ligands **4a**, **4b**, and **5a** have cytostatic properties on a larger set of neoplasia cell models. For the assay, 1.5 × 10^3^ Capan2, 4 × 10^3^ U2OS, or 5 × 10^3^ L428 cells were plated into 96-well plates. Capan2 and U2OS cells were treated with the compounds in the concentrations indicated for 48 h, and then an SRB assay was performed. L428 cells were treated with the compounds in the concentrations indicated for 96 h, and then the cells were counted using a Bürker chamber. Data are represented as average ± SD from three biological replicates; individual assays were performed in duplicate. Values were normalized for vehicle-treated cells, and the absorbance for vehicle-treated cells equals 1. Normality was assessed using the Shapiro–Wilk test. Statistical significance was assessed using one-way ANOVA or Kruskal–Wallis test as a function of normality, followed by Holm–Sidak’s, Dunnett’s, or Dunn’s post hoc test, respectively. For better visibility, normality, transformations, statistical tests, and p values are presented in an MS Excel sheet at https://figshare.com/s/3a0aa60b66f3f746f41e. Nonlinear regression was performed on the datasets; IC_50_ values are indicated in Table 5. Color code: khaki—ruthenium complex (**Ru-4a,b**), blue—osmium complex (**Os-4a,b** or **Os-5a**), green—iridium complex (**Ir-4a,b**), and red—rhodium complex (**Rh-4a,b**).

Next, we investigated whether the bioactive complexes would be active against a larger set of neoplasias. This set of cells was selected to include another carcinoma (Capan2, a pancreatic adenocarcinoma), a sarcoma (U2OS, an osteosarcoma), and a lymphoma (L428, a Hodgkin lymphoma) cell line. Similar to the results on ovarian cancer cell lines, the complexes previously identified to possess cytostatic activity (**Ru/Os/Ir-4a**,**b**, **Os-5a**) proved to be active on these models, too (Figure 8). Another important difference between the activity of the complexes on A2780 and ID8 cells versus on Capan-2 and U2OS cells was the narrowing of the therapeutic index (IC_50_ value on the cancer cell line vs IC_50_ value on primary human dermal fibroblasts), likely limiting the possible use of these complexes in diseases other than ovarian cancer.

#### 2.4.4 The bioactive complexes have cytostatic properties on cisplatin-resistant A2780 cells

Cisplatin-resistance is a factor frequently limiting the complete delivery of platinum-based therapy (McMullen et al., 2021; Sipos et al., 2021); therefore, we assessed whether these complexes can induce cytostasis in cisplatin-resistant A2780 cells. The cisplatin-resistant cells were purchased from Sigma-Aldrich, and we characterized these cells in one of our previous studies (Kacsir et al., 2023a). The difference in the IC_50_ values of the cisplatin-sensitive versus the cisplatin-resistant cells to cisplatin was 13.6-fold (1.21 µM → 16.47 µM). Rhodium complexes were excluded from the investigation as their activity on A2780 cells was negligible (Figure 7; Table 5).

**FIGURE 9:**
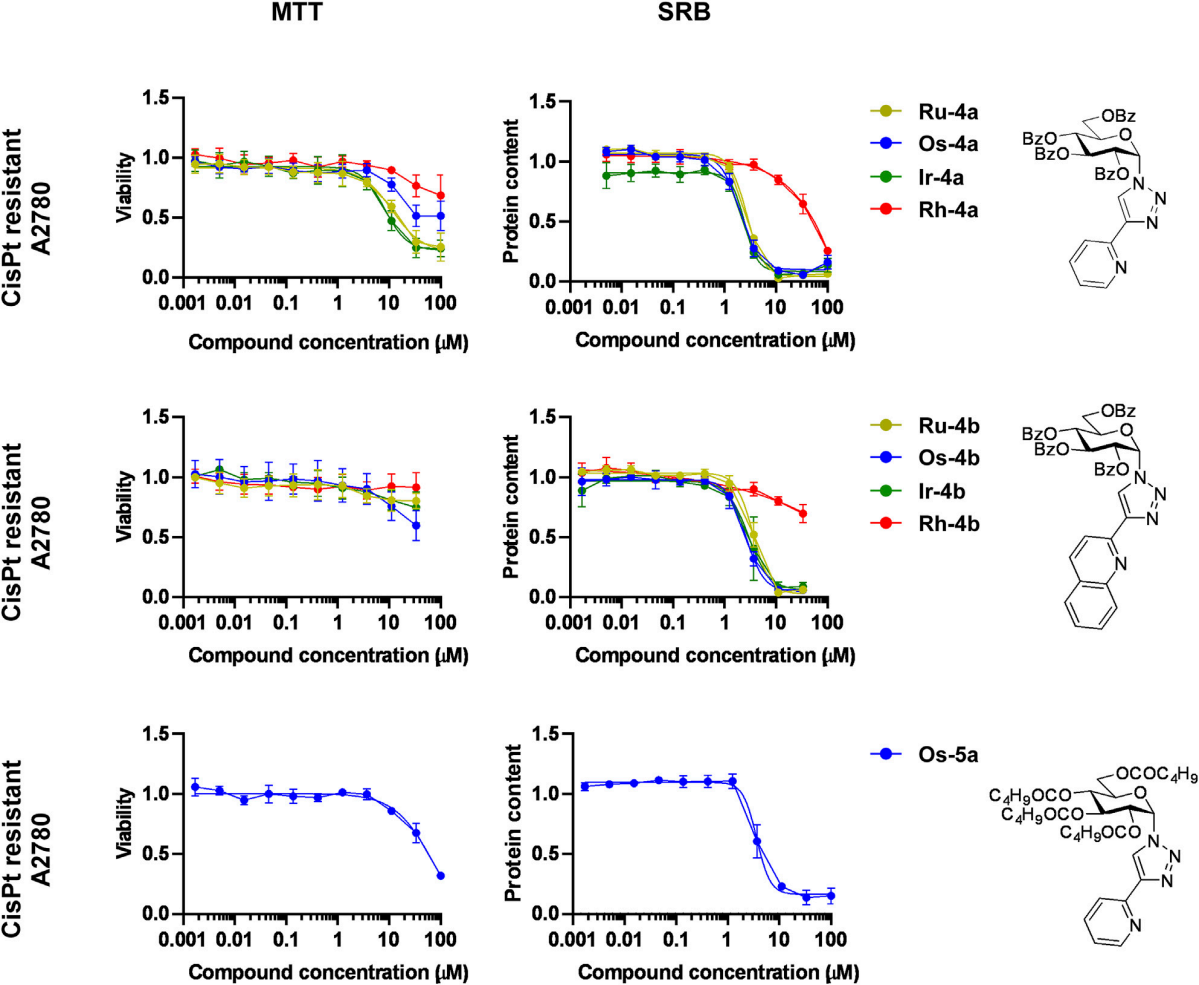
The complexes of ligands **4a**, **4b**, and **5a** have cytostatic properties on cisplatin-resistant A2780 cells. For MTT assays, 6.5 × 10^3^ cisplatin-resistant A2780 cells were plated into 96-well plates, and for SRB assays, 4 × 10^3^ cisplatin-resistant A2780 cells were plated into 96-well plates. Cells were treated with the compounds in the concentrations indicated for either 4 h for an MTT assay or 48 h for an SRB assay. The n values for the assays are the following: n = 3 for **Ru/Os-4a** in SRB, **Rh-4a**, **Ru/Ir-4b**, and **Os-5a** in MTT and SRB; n = 4 for **Ir-4a**, **Os/Rh-4b** in MTT and SRB; n = 6 for **Ru/Os-4a** in MTT. Data are represented as average ± SD, and individual assays were performed in duplicate. Values were normalized for vehicle-treated cells, and the absorbance for vehicle-treated cells equals 1. Normality was assessed using the Shapiro–Wilk test. Statistical significance was assessed using one-way ANOVA or Kruskal–Wallis test as a function of normality, followed by Holm–Sidak’s, Dunnett’s, or Dunn’s *post hoc* test. For better visibility, normality, transformations, statistical tests, and p values are presented in an MS Excel sheet at https://figshare.com/s/3a0aa60b66f3f746f41e. Nonlinear regression was performed on the datasets; IC_50_ values are indicated in Table 5. Color code: khaki—ruthenium complex (**Ru-4a,b**), blue—osmium complex (**Os-4a,b** or **Os-5a**), green—iridium complex (**Ir-4a,b**), and red—rhodium complex (**Rh-4a,b**).

The ruthenium(II), osmium(II), and iridium(III) complexes of **4a**, **4b**, and **5a** exerted cytostatic activity on the cisplatin-resistant A2780 cells with somewhat elevated IC_50_ values that were similar to the IC_50_ values of the cisplatin-sensitive A2780 cells (Figure 9; Table 5). With regards to the MTT assays, the ruthenium(II), osmium(II) and iridium(III) complexes of **4a**, **4b**, **5a** did not affect MTT reduction (Figure 9; Table 5), in contrast to their effects on A2780 cells or human primary fibroblasts (Figure 7; Table 5).

#### 2.4.5 Bioactive complexes exert cytostasis through inducing oxidative stress

**FIGURE 10:**
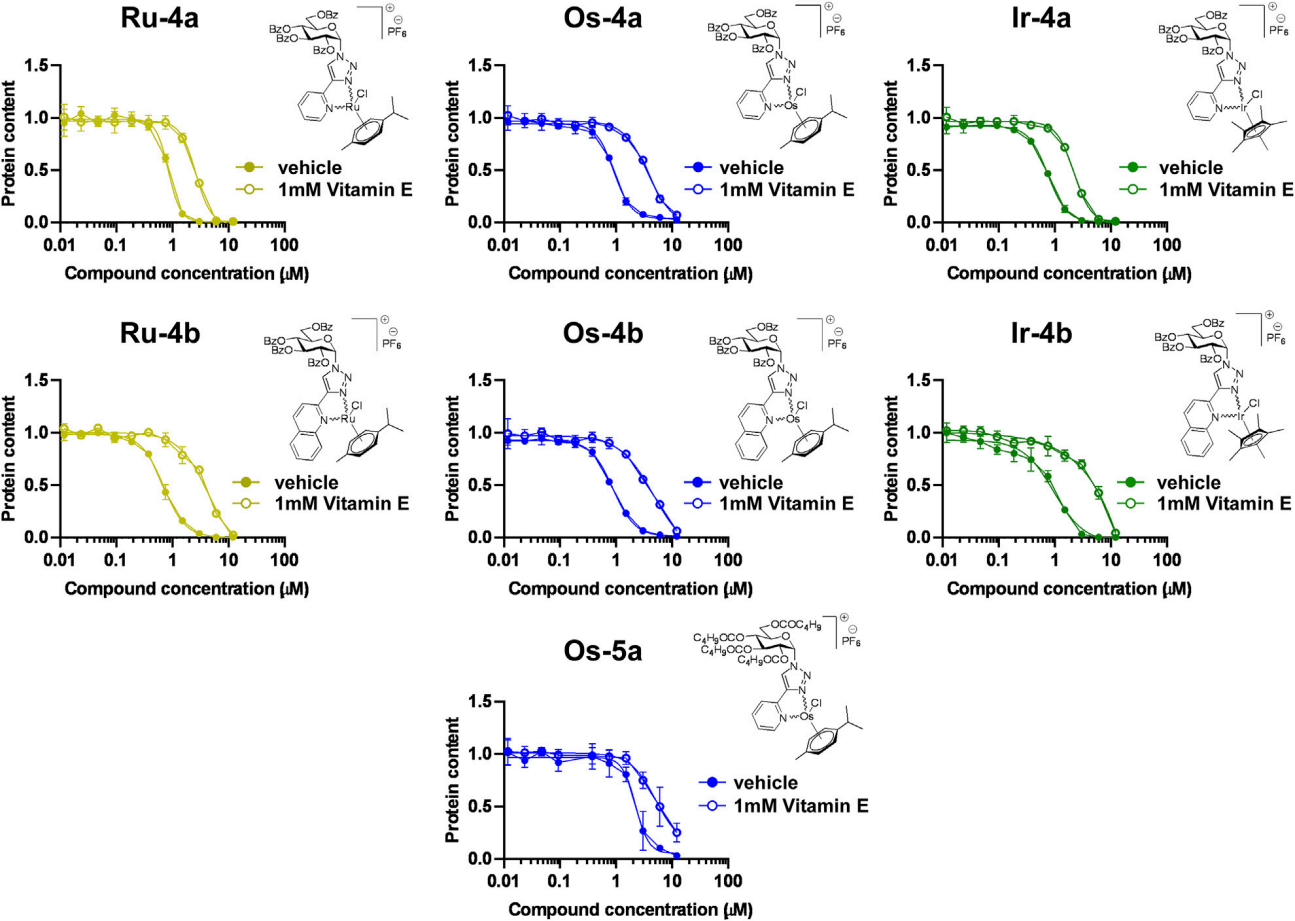
The cytostatic effects of the complexes of ligands **4a**, **4b**, and **5a** are alleviated by vitamin E. 1.5 × 10^3^ A2780 cells were plated into 96-well plates. Cells were treated with the compounds in the concentrations indicated with or without vitamin E for 48 h, followed by an SRB assay. Data are represented as average ± SD, from three biological replicates. Values were normalized for vehicle-treated cells, and the absorbance for vehicle-treated cells equals 1. Data are represented as fold change compared to vehicle-treated controls. Normality was assessed using the D’Agostino–Pearson test. Statistical significance was assessed using a two-way ANOVA test followed by Tukey’s post hoc test. Nonlinear regression was performed on the datasets. For better visibility, normality, transformations, statistical tests, IC_50_ values, and p values are presented in an MS Excel sheet at https://figshare.com/s/3a0aa60b66f3f746f41e. Color code: khaki—ruthenium complex (**Ru-4a,b**), blue—osmium complex (**Os-4a,b** or **Os-5a**), and green—iridium complex (**Ir-4a,b**).

Previous data evidenced that complexes with a similar structure induce oxidative stress in mammalian cells and in bacteria (Xu et al., 2018; Fernandes, 2019; Bakewell et al., 2020; Mihajlovic et al., 2020; Kacsir et al., 2021; Kacsir et al., 2022; Kacsir et al., 2023a; Kacsir et al., 2023b), suggesting that the α-anomer complexes discussed in the current manuscript may also act through eliciting oxidative stress. To study that, we applied vitamin E to scavenge reactive oxygen species (ROS). Vitamin E treatment led to a rightward shift in the sigmoid inhibitory curve of the complexes **Ru/Os/Ir-4a**,**b, Os-5a** (Figure 10), among which only **Ru-4a**, **Os-4b** showed a trend but lacked statistical significance. These observations suggest that ROS scavenging by vitamin E protects against the cytostatic property of the complexes. Vitamin E has an apolar phytyl chain, rendering the whole molecule apolar and prone to protect biomembranes from oxidation (Hon-Wing et al., 1981). Trolox, a version of vitamin E without the apolar phytyl chain, does not have a protective effect on cells treated with the complexes of the same structure but with a β-anomeric glucose moiety (Kacsir et al., 2021). These suggest that the complexes likely induce the oxidation of biomembranes and/or elicit pathways that stem from oxidized biomembranes (e.g., chaperone-induction (Horváth et al., 1998; Gombos et al., 2011).

#### 2.4.6 Bioactive ruthenium(II), osmium(II), and iridium(III) complexes have limited bacteriostatic activity

Previously, we showed that other half-sandwich complexes with a similar structure have bacteriostatic properties on sensitive and multiresistant isolates of Gram-positive species such as *Staphylococcus aureus* and *Enterococcus faecalis* or *E. faecium* (Balázs et al., 2022; Kacsir et al., 2023a; Kacsir et al., 2023b). Along the same lines, we tested the bioactive members of the newly prepared complexes on a reference strain and clinical isolates of *Staphylococcus aureus* and *Enterococcus faecium*. Our earlier observation for analogous complexes was that only those complexes that possessed antibacterial features which had cytostatic properties on mammalian cancer cells. For that reason, we applied only those complexes of the new set that were cytostatic on A2780 cells, namely, **Ru/Os/Ir-4a**,**b** and **Os-5a**.

**FIGURE 11:**
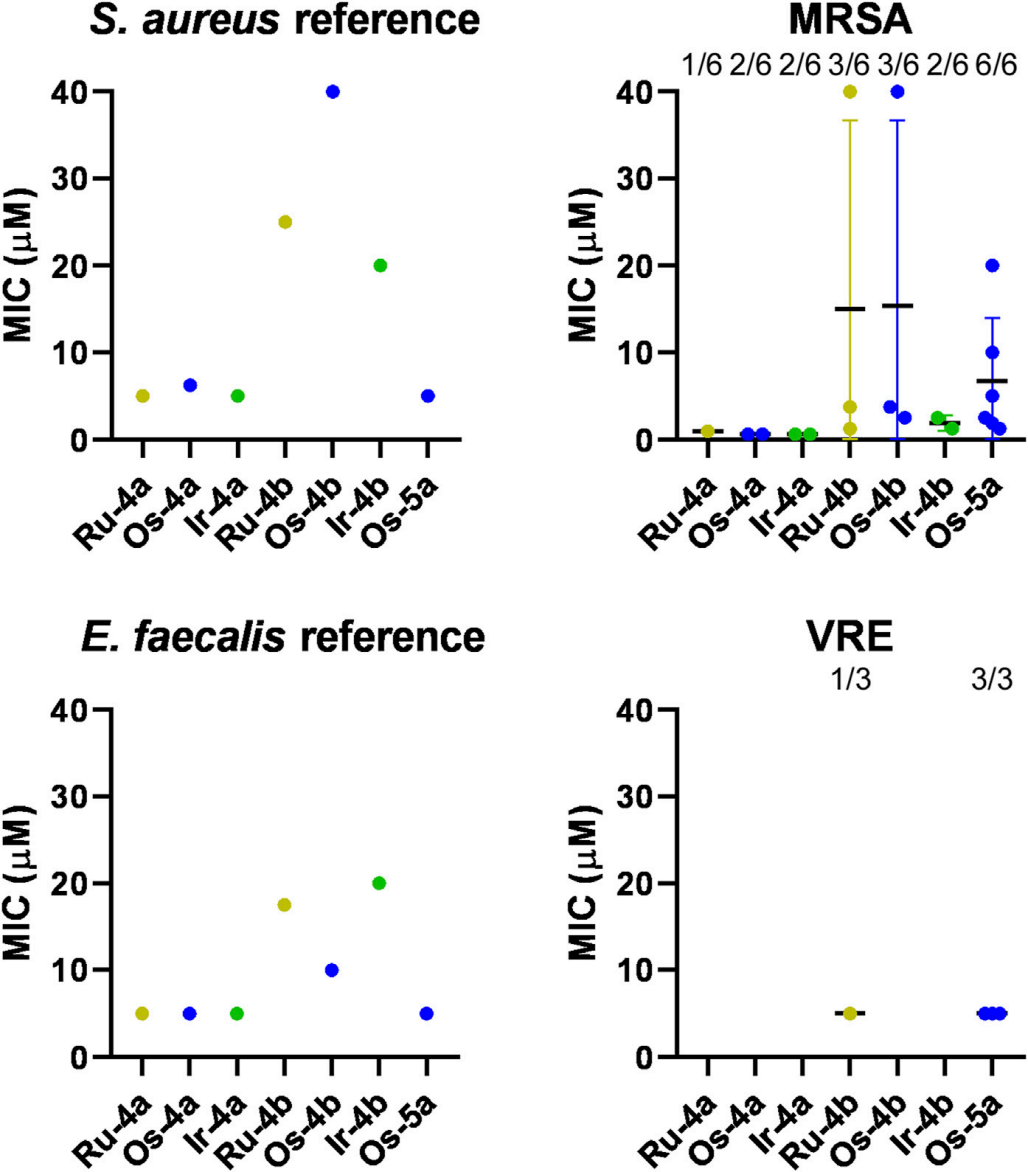
The bacteriostatic activity of the bioactive complexes on MRSA and VRE isolates. The MIC values of the complexes were determined against the reference strains of *S. aureus* (ATCC 29213) and *E. faecalis* (ATCC 29212) and clinical VRE and MRSA isolates by microdilution assays (repeated at least twice in duplicate) as described in Materials and Methods. The numbers indicate how many isolates were susceptible to the compound of those tested; that is, 1/6 indicates that one isolate was susceptible of six tested. Abbreviations: MRSA, methicillin-resistant *Staphylococcus aureus*; VRE, vancomycin-resistant *Enterococcus*. Color code: khaki—ruthenium complex (**Ru-4a,b**), blue—osmium complex (**Os-4a,b** or **Os-5a**), and green—iridium complex (**Ir-4a,b**).

The MIC values determined for the reference strain of *S. aureus* were in the range of 5–40 μM, whereas those for *E. faecalis* were in the range of 5–20 µM, and both reference strains were sensitive to all of the studied complexes (Figure 11; Table 6). In contrast, not all clinical isolates were sensitive to all complexes. In case of the MRSA and VRE clinical isolates, complexes with *O*-perbenzoylated sugar moiety (**Ru/Os/Ir-4a**,**b**) were not bacteriostatic on all isolates in contrast to **Os-5a**, which has a pentanoylated sugar unit (Figure 11; Table 6). The MIC values of the effective complexes were similar to or better than the MIC values on the reference strains (Figure 11; Table 6).

**TABLE 6:** The clinical isolates used in the study and the respective MIC values of the complexes on those isolates. Abbreviations: VRE—vancomycin-resistant *Enterococcus*, MRSA—methicillin-resistant *Staphylococcus aureus*.

Species and Strain	Identifier	Sample	Year	MIC (µM)						
				**Ru-4a**	**Os-4a**	**Ir-4a**	**Os-4b**	**Ir-4b**	**Ru-4b**	**Os-5a**
Reference *E. faecalis*				5	5	5	10	20	17.5	5
VRE	25,051	Nephrostoma	2018	>40	>40	>40	>40	>40	>40	5
	27,085	Wound	2018	>40	>40	>40	>40	>40	5	5
	25,498	Rectal swab to screen for multiresistant pathogens	2018	>40	>40	>40	>40	>40	>40	5
Reference *S. aureus*				5	6.25	5	40	20	25	5
MRSA	24,272	Throat	2018	0.938	0.625	0.625	2.5	2.5	1.25	1.88
	24,408	Bronchial	2018	>40	>40	>40	40	>40	40	2.5
	20,426	Blood	2020	>40	>40	>40	>40	>40	>40	5
	24,035	Wound	2018	>40	>40	>40	>40	>40	>40	1.25
	24,328	Throat	2018	>40	0.625	0.625	3.75	1.25	3.75	20
	24,268	Throat	2018	>40	>40	>40	>40	>40	>40	10

## 3 Discussion

In this study, we assessed the antineoplastic and antimicrobial potential of platinum-group metal half-sandwich complexes of α-d-glucopyranosyl 1,2,3-triazole-type ligands. The most important observations demonstrated that 1) the lipophilic character of the complexes, triggered by suitable *O*-protection of the sugar moiety, is a prerequisite for their biological activity: complexes of the unprotected and *O*-peracetylated glucose-derived ligands **2a,b** and **3a** with negative logD values were ineffective, while the use of larger, aromatic (benzoyl) or open-chain (pentanoyl) acyl protection in the sugar part (**4a,b**, **5a**) induced positive logD values, thereby rendering most of the respective complexes to be bioactive; 2) complexes with 2-quinolyl substituted 1,2,3-triazole ligands (**b**) displayed better effects than those with 2-pyridyl substituents (**a**); 3) the compounds showed widespread activity among cancer cell lines; 4) cooperative binding of the complexes took place to their targets reflected by Hill coefficients larger than 1; 5) *p*-cym-Ru(II), *p*-cym-Os(II), and Cp*-Ir(III) complexes possessed bioactivity in contrast to Cp*-Rh(III) derivatives, and the *p*-cym-Os(II) complexes with the lowest IC_50_ values on cancer cells were the most efficient members of the set. Although complexes with slightly lower tendency for hydrolysis were detected in the organorhodium-**3a** system than in the organoruthenium system, as indicated by the solution equilibrium study, inactivity of **Rh-3a** can be explained by the rather labile character of this complex compared to those formed with the other three metal ions.

In terms of biological effects, the α-anomer-containing complexes (α-complexes hereafter) phenocopied certain features of the previously described β-anomer-containing complexes (Kacsir et al., 2021; Kacsir et al., 2022; Kacsir et al., 2023a) (β-complexes hereafter) in terms of 1) selectivity toward cancer cells, 2) the induction of oxidative stress to induce cytostasis or bacteriostasis, and 3) effectiveness on cisplatin-resistant cells. On mammalian cells, the α-complexes had similar potency (i.e., similar IC_50_ values) as the β-complexes. Furthermore, the pattern of the IC_50_ values was also similar between the α- and β-complexes, with the lowest IC_50_ values on A2780 and ID8 cells, somewhat higher IC_50_ values on other cancer cell lines and an at least ten-fold larger IC_50_ on human primary dermal fibroblasts. The lowest IC_50_ value among the complexes described in this report on A2780 cells is submicromolar (0.65 µM for **Os-4b**), with a corresponding IC_50_ value on primary human dermal fibroblasts of 10.54 µM. The corresponding β-complex (Kacsir et al., 2023a) had an IC_50_ value of 0.58 µM on A2780 cells, and the IC_50_ value was not detectable on primary human dermal fibroblasts.

Although the α- and β-complexes had similar behavior on mammalian cells, marked differences were observed with regard to their bacteriostatic capacity. Namely, the α-complexes with *O*-perbenzoylated glucose units were active only on a subset of the MRSA isolates and were largely inactive on VRE isolates. This was unexpected, as in previous studies, β-complexes with similar structural components were active on all of the MRSA and VRE isolates used in our current study (Balázs et al., 2022; Kacsir et al., 2023a; Kacsir et al., 2023b). Only one complex, **Os-5a** from the present study, was bacteriostatic on all MRSA and VRE isolates with low micromolar average MIC values (6.8 µM on MRSA and 5 µM on VRE isolates). Interestingly, bacteriostatic complexes had lower IC_50_ values on mammalian cells than their MIC values on bacteria. The MIC value of **Os-5a** is comparable to the MIC values of the previously identified bacteriostatic complexes (Balázs et al., 2022; Kacsir et al., 2023a; Kacsir et al., 2023b). Of note, a limitation of our study is the low number of clinical isolates.

There are literature reports for the antibacterial activity of piano stool complexes of ruthenium, rhodium, iridium, and osmium on *Mycobacterium* species (Karpin et al., 2013; DuChane et al., 2018; Yufanyi et al., 2020; Bernier et al., 2021; Coverdale et al., 2021), *Klebsiella pneumoniae*, *Escherichia coli*, and *S. aureus* (Lapasam et al., 2020a; Lapasam et al., 2020c). The inhibitory properties of the complexes cannot be directly compared to our results in the case of each study as different model systems are used in certain reports (i.e., disc diffusion test *versus* MIC value determination), nevertheless, for the compatible studies, Os(II) complexes had comparable or superior MIC values on *Mycobacterium* ((Coverdale et al., 2021) 1.25–2.5 µM; (Bernier et al., 2021); best MIC value 0.45 µM). Unfortunately, the inhibitory values for *Staphylococcus aureus* (Lapasam et al., 2020a; Lapasam et al., 2020c) are not comparable due to the aforementioned technical differences. Of note, a bacteriostatic property is not a common trait for the half-sandwich complexes with a similar structure to those assessed in this study (Pivarcsik et al., 2022) vs. (Balázs et al., 2022; Kacsir et al., 2023a; Kacsir et al., 2023b).

Taken together, the change of the configuration of the C1 carbon of the carbohydrate moiety from β to α did not largely affect the antineoplastic activity of the complexes. However, the α-complexes had limited bacteriostatic activity, and only the complex with pentanoyl protective groups on the carbohydrate moiety exerted bacteriostatic activity. Our study suggests that while similar structural components render half-sandwich complexes cytostatic and bacteriostatic, different structural modifications are necessary for bacteriostatic activity or cytostatic activity; hence, complexes must be fine-tuned as a function of the intended use.

## 4 Materials and Methods

### 4.1 Syntheses

#### 4.1.1 General methods

Optical rotation measurements were conducted on a Jasco P-2000 polarimeter (Jasco, Easton, MD, USA) at ambient temperature, with reported values representing the average of three parallel determinations. NMR spectra were recorded using Bruker (Karlsruhe, Germany) spectrometers: DRX360 (360/90 MHz for ^1^H/^13^C), DRX400 (400/100 MHz for ^1^H/^13^C), and Avance II 500 (500/125 MHz for ^1^H/^13^C). Me_4_Si was applied as the reference for chemical shifts of ^1^H-NMR, while the residual solvent signals were used for those of ^13^C-NMR. The proton- and carbon-signal assignments for characteristic resonances of the prepared complexes were based on COSY and HSQC correlations of some representatives of the series (**Ru-2b**, **Ir-2b**, **Os-4b**, **Rh-4b**). ESI-HRMS data were obtained by measurements on a Bruker maXis II spectrometer using positive ionization mode. TLC analyses were conducted on DC Kieselgel 60 F_254_ plates (Sigma-Aldrich), with visualization achieved under UV light or by gentle heating. For purifications carried out by column chromatography, Kieselgel 60 silica gel (particle size 0.063–0.2 mm, Molar Chemicals) was used as the stationary phase. Among the anhydrous solvents used, pyridine was acquired from VWR Chemicals, while the others were prepared in our laboratory following established distillation protocols: halogenated solvents (CH_2_Cl_2_ and CHCl_3_) were distilled from P_4_O_10_ and stored over 4 Å molecular sieves, and methanol was dried by distillation over magnesium turnings and iodine. 2-Ethynylpyridine (TCI Chemicals), pentanoyl chloride (Alfa Aesar), TlPF_6_ (Strem Chemicals), dichloro (η^6^-*p*-cymene)ruthenium(II) dimer (**Ru-dimer**, Strem Chemicals), dichloro (η^5^-pentamethylcyclopentadienyl)iridium(III) dimer (**Ir-dimer**, Acros Organics), and dichloro (η^5^-pentamethylcyclopentadienyl)rhodium(III) dimer (**Rh-dimer**, Alfa Aesar) were purchased from the given suppliers. 2,3,4,6-Tetra-*O*-acetyl-α-d-glucopyranosyl azide (Zhang et al., 1999) (**1**), 2-ethynylquinoline (Son et al., 2013), and dichloro (η^6^-*p*-cymene)osmium(II) dimer (Godó et al., 2012) (**Os-dimer**) were synthesized in accordance with literature methods.

#### 4.1.2 General procedure I for the synthesis of 1-(2′,3′,4′,6′-tetra-*O*-acetyl-α-d-glucopyranosyl)-4-hetaryl-1,2,3-triazoles

2,3,4,6-Tetra-*O*-acetyl-α-d-glucopyranosyl azide (Zhang et al., 1999) (**1**) was dissolved in a solvent mixture of *t*-BuOH-H_2_O (15–15 mL/1 g of azide). To this solution, the corresponding 2-ethynylated heterocycle (1.4 equiv.), l-ascorbic acid (0.8 equiv.) and CuSO_4_·5H_2_O (0.2 equiv.) were added. The reaction mixture was heated at 70°C under stirring. When the TLC (1:1 EtOAc-hexane) showed complete disappearance of the starting azide (∼1 day), the reaction mixture was diluted with water (30 mL) and extracted with CH_2_Cl_2_ (3 × 50 mL). The organic layers were combined and washed with 5% EDTA in 1 M aqueous solution of NH_4_OH (30 mL), then with water (50 mL). The separated organic phase was dried over anhydrous MgSO_4_, filtered, and evaporated under diminished pressure. Purification of the residual crude product was carried out by column chromatography.

#### 4.1.3 General procedure II for the deacetylation of the 1-(2′,3′,4′,6′-tetra-*O*-acetyl-α-d-glucopyranosyl)-4-hetaryl-1,2,3-triazoles by the Zemplén method

The appropriate *O*-peracetylated 1-(2′,3′,4′,6′-tetra-*O*-acetyl-α-d-glucopyranosyl)-4-hetaryl-1,2,3-triazole (**2a,b**) was dissolved in a solvent mixture of anhydrous methanol and anhydrous chloroform (2–2 mL/100 mg of triazole). To this solution, a few drops of a 1 M solution of sodium methoxide in methanol were added to adjust the pH to a range of 8–9. The reaction mixture was left to stand at ambient temperature, and the transformation was monitored by TLC (1:1 EtOAc-hexane and 7:2 CHCl_3_-MeOH). After completion of the reaction, the mixture was treated with a cation exchange resin (Amberlyst 15, in H^+^ form) for neutralization. Subsequently, the resin was removed by filtration, and the solution was concentrated under reduced pressure. The pure compound was obtained by column chromatographic purification.

#### 4.1.4 General procedure III for the *O*-peracylation of the 1-(α-d-glucopyranosyl)-4-hetaryl-1,2,3-triazoles

The corresponding 1-(α-d-glucopyranosyl)-4-hetaryl-1,2,3-triazole (**3a,b**) was dissolved in anhydrous pyridine (4 mL/50 mg triazole). To this solution, the appropriate carboxylic acid chloride (4.8 equiv.) was added under stirring. The reaction mixture was subsequently heated to 60°C, and the transformation was monitored by TLC (7:2 CHCl_3_-MeOH and 1:2 EtOAc-hexane). After 2 h, the TLC indicated incompleteness of the reaction; therefore, additional portions of acid chloride (2 × 4.8 equiv. per 4 h) were added to the mixture, and then the stirring was continued at 60°C overnight. After that, the pyridine was removed under reduced pressure, and the residue was diluted with water (20 mL) and extracted with CHCl_3_ (2 × 20 mL). The combined organic layers were extracted with a saturated aqueous solution of NaHCO_3_ (3 × 20 mL), then with water (20 mL). The organic layer was dried (MgSO_4_), filtered, and the solvent was removed *in vacuo*. The target pure compound was obtained by column chromatographic purification of the residue.

#### 4.1.5 General procedure IV for the synthesis of the half-sandwich platinum-group metal complexes of the *O*-peracylated and *O*-unprotected 1-(α-d-glucopyranosyl)-4-hetaryl-1,2,3-triazoles

To a solution of the appropriate dimeric chloro-bridged metal complex (**Ru-dimer/Os-dimer/Ir-dimer**/**Rh-dimer**) in anhydrous CH_2_Cl_2_ (1 mL/10 mg dimer), the corresponding 1-(α-d-glucopyranosyl)-4-hetaryl-1,2,3-triazole (2.0–2.3 equiv.) and TlPF_6_ (2 equiv.) were added. Under stirring at ambient temperature, anhydrous MeOH (1 mL/10 mg dimer) was also added to the reaction mixture to promote the precipitation of the TlCl. The stirring was continued at the same temperature until the TLC (95:5 CHCl_3_-MeOH) indicated the total consumption of the dimer (∼1 h). The TlCl was removed by filtration using a syringe filter (Nylon, 25 mm, 0.22 µm), and the resulting solution was evaporated *in vacuo*. The pure complex from the residue was obtained by trituration in a solvent mixture, recrystallization, or column chromatographic purification.

### 4.2 Determination of the distribution coefficients (logD)

The distribution coefficient (logD) of the new complexes was determined using a 1:1 mixture of *n*-octanol-aq. PBS solution (pH = 7.4) according to our earlier published procedure (Kacsir et al., 2021).

### 4.3 Solution equilibrium studies

For solution studies, doubly deionized and ultra-filtered water was obtained from a Milli-Q RG (Millipore) water purification system. pH-potentiometric measurements were carried out at a constant ionic strength of 0.20 M KCl and at 25.0°C. Carbonate-free KOH solutions of known concentrations (ca. 0.2 M) were used as titrant. HCl stock solutions were prepared from concentrated HCl, respectively, and their concentrations were determined by potentiometric titrations using Gran’s method (Gran et al., 1950). A Mettler Toledo DL50 titrator equipped with a DG114-SC combined glass electrode was used for the pH-potentiometric measurements. The electrode systems were calibrated according to Irving et al. (1967); the pH-metric readings could therefore be converted into hydrogen ion concentration. The water ionization constant, p*K*_w_, was 13.74 ± 0.01 under the conditions employed. The initial volume of the samples was 15.00 mL. The metal ion concentrations were varied in the range 0.9–1.8 mM. The samples were in all cases completely deoxygenated by bubbling purified nitrogen for ca. 20 min before the measurements. The titrations were performed in the pH range of 2.0–11.0 in equilibrium-controlled mode, during which the pH equilibrium was assumed to be reached if a change in the measured potential was less than 0.1 mV within 90 s. The minimum waiting time was 1.5 min, while the maximum was up to 10 min. The protonation constants of the ligands and the overall stability constants of the complexes, β_p,q,r_ = [M_p_H_q_L_r_]/[M]^p^[H]^q^[L]^r^ (where “M” stands for [(η^5^-Cp*)Rh(H_2_O)_3_]^2+^ and “L” represents the completely deprotonated form of the ligand), were calculated with the aid of the SUPERQUAD (Gans et al., 1985) and PSEQUAD (Zékány and Nagypál, 1985) computer programs, respectively. During the calculations, hydrolysis of the metal ions was taken into consideration. The stability constants of the hydroxido complexes in chloride-containing medium involved in the equilibrium models were taken from the literature (Bíró et al., 2012; Bíró et al., 2013; Dömötör et al., 2014).

### 4.4 Chemicals for biology experiments

All chemicals used in the cell biology and biochemistry assays were obtained from Sigma-Aldrich unless otherwise stated. The free ligands and complexes investigated in this study were dissolved in dimethylsulfoxide for biology experiments, and 0.1% dimethylsulfoxide was used as a vehicle control.

### 4.5 Cell culture

Cells were cultured under standard cell culture conditions: 37°C, 5% CO_2_, humidified atmosphere. *A2780* cells were cultured in RPMI 1640 medium, supplemented with 10% fetal calf serum, 2 mM glutamine, and 1% penicillin–streptomycin.

*ID8* cells were cultured in a high-glucose DMEM (4.5 g/L glucose) medium, supplemented with 4% fetal calf serum, 2 mM glutamine, 1% penicillin–streptomycin, and 1% ITS supplement (I3146).

*Capan2* cells were maintained in MEM, 10% fetal bovine serum, 1% penicillin–streptomycin, and 2 mM glutamine.

*Human primary dermal fibroblasts* were cultured in low-glucose DMEM (1 g/L glucose) medium supplemented with 20% fetal calf serum, 2 mM glutamine, and 1% penicillin–streptomycin.

*L428* cells were maintained in RPMI 1640 medium, supplemented with 10% fetal calf serum, 2 mM glutamine, and 1% penicillin–streptomycin.

*U2OS* cells were maintained in high-glucose DMEM (4.5 g/L glucose) medium, supplemented with 10% fetal calf serum, 2 mM glutamine, and 1% penicillin–streptomycin.

### 4.6 Bacterial reference strains

The reference strains of *Staphylococcus aureus* (ATCC 29213) and *Enterococcus faecalis* (ATCC 29212) were purchased from the ATCC (Manassas, VA, United States).

### 4.7 Clinical isolates of *S. aureus* and *E. faecium*

We used a set of clinical isolates of *S. aureus* and *E. faecium* that were collected at the Medical Center of the University of Debrecen (Hungary) between 1 January 2018 and 31 December 2020. The isolates were reported in Balázs et al. (2022) and are presented in Table 6. The clinical isolates were identified using a Microflex MALDI-TOF mass spectrometer (Bruker, Billerica, MA, United States). The antibiotic susceptibility of the isolates was tested following the European Committee on Antimicrobial Susceptibility Testing (EUCAST) guidelines, which were valid at the time of collection.

### 4.8 Broth microdilution

Microdilution experiments were performed according to the standards of EUCAST (EUCAST, 2025). The bacterial isolates to be tested were grown on Mueller–Hinton agar plates. The inoculum density of bacteria was set at 5.0 × 10^5^ CFU/mL in microtiter plates in a final volume of 200 µL Mueller–Hinton broth. The tested concentration range was 0.08–40 µM (10 concentrations, two-fold serial dilutions), and a drug-free growth control and an inoculum-free negative control were included. The inoculated plates were incubated for 24 h at 37°C, then visually assessed. Minimum inhibitory concentration (MIC) was defined as the lowest concentration with inhibitory effect compared to the growth control. All experiments were performed at least twice in duplicate.

### 4.9 Methylthiazolyldiphenyl-tetrazolium bromide (MTT) reduction assay

An MTT reduction assay measures the activity of mitochondrial complex I and can be used to detect toxicity (Henslee et al., 2016). The assay was performed in a manner similar to that described by Kacsir et al. (2023b). Briefly, cells were plated into 96-well plates the day before the assay. Cells were treated with the compounds for 4 h; then, MTT was added to a 0.5 mg/mL final concentration, and cells were incubated at 37°C in a cell incubator for 40–60 min, as a function of the cell line being assessed. The culture medium was removed, the reduced MTT dye was dissolved in dimethylsulfoxide, and plates were measured in a plate photometer (Thermo Scientific Multiscan GO spectrophotometer, Waltham, MA, United States) at 540 nm. Certain wells were designated to contain vehicle-treated cells on each plate. In calculations, the readings for these wells were considered to 1, and all readings were expressed relative to these values.

### 4.10 Sulforhodamine B (SRB) binding assay

An SRB assay measures protein content of cells in correlation with the cell number in an assay well and can therefore be used to assess cell proliferation or long-term cytostasis (Skehan et al., 1990). Cells were seeded into 96-well plates the day before the treatment for the assay. Cells were treated with the various compounds for 48 h. Then, the medium was removed, and cells were fixed with 10% trichloroacetic acid. Fixed cells were washed in distilled water three times, followed by staining with SRB (0.4 m/V% dissolved in 1% acetic acid) for 10 min. Stained cells were washed in 1% acetic acid five times; the acetic acid was removed, and the cells were left to dry. Protein-bound SRB was released by adding 100 µL of 10 mM Tris base. Plates were measured in a plate photometer (Thermo Scientific Multiscan GO spectrophotometer, Waltham, MA, United States) at 540 nm. Certain wells were designated to contain vehicle-treated cells on each plate. In calculations, the readings for these wells were considered to be 1, and all readings were expressed relative to these values.

### 4.11 Annexin V–propidium iodide staining for the determination of cell death

The proportion of dead cells was assessed using the annexin V–propidium iodide assay and was measured using flow cytometry with an ACEA NovoCyte 3000 Flow Cytometer (Agilent Technologies, Santa Clara, CA, United States) instrument and the FITC annexin V/Dead Cell Apoptosis kit (Life Technologies, Eugene, OR, USA), according to the manufacturer’s instructions, in a process similar to that described by Bai et al. (2001). Quadrants were set based on the FITC and propidium iodide (PI) values observed for the vehicle-treated cells. Double negative cells were considered living cells, and the other three quadrants were considered cells in different modes and phases of cell death and were added up. Heat-shocked cells were used as positive controls (30 min at 42°C in Eppendorf tubes similar to Nagy et al. (2007).

### 4.12 Statistical evaluation

Statistical analysis was performed using version 8.0.1 of GraphPad Prism. Values were tested for normal distribution using the D’Agostino–Pearson or Shapiro–Wilk normality tests. When necessary, values were log-normalized or normalized using the Box–Cox or two-step normalization method (Box and Cox, 1964), as indicated in the MS Excel file listing the values at https://figshare.com/s/3a0aa60b66f3f746f41e. The following statistical test, *post hoc* test, and the level of significance are indicated in the MS Excel file listing the values at https://figshare.com/s/3a0aa60b66f3f746f41e. Nonlinear regression was performed using the built-in “[Inhibitor] vs response—Variable slope (four parameters), least square fit” utility of GraphPad, which yielded IC_50_ and Hill slope values if the sigmoid curves reached a plateau of inhibition, and there was no decrease between two subsequent data points or when inhibition was over 90%. In other cases, the percentage of inhibition was taken for the maximum concentration (100 µM).

## Data Availability

The datasets presented in the study can be found here: https://figshare.com/s/3a0aa60b66f3f746f41e.

## Footnotes

1 These data can be obtained free from www.ccdc.cam.ac.uk/data_request/cif, or by emailing data_request@ccdc.cam.ac.uk, or by contacting The Cambridge Crystallographic Data Centre, 12 Union Road, Cambridge CB2 1EZ, UK; fax: +44 1223 336033.
