# Supplementary material for: Platinum-group metal half-sandwich complexes of 1-(α-d-glucopyranosyl)-4-hetaryl-1,2,3-triazoles: synthesis, solution equilibrium studies, and investigation of their anticancer and antimicrobial activities
Source: Front Chem. 2025 Sep 1;13:1619991. doi: 10.3389/fchem.2025.1619991 (PMC12434071; doi:10.3389/fchem.2025.1619991)
Supplement: Supplementary file 1 [file DataSheet1.docx]

Supplementary Material

**Table of contents**

[1 Synthetic details 3](#_Toc196488100)

[1.1 General methods 3](#_Toc196488101)

[1.2 General procedure I for the synthesis of 1-(2’,3’,4’,6’-tetra-*O*-acetyl-α-D-glucopyranosyl)-
4-hetaryl-1,2,3-triazoles 3](#_Toc196488102)

[1.3 General procedure II for the deacetylation of the 1-(2’,3’,4’,6’-tetra-*O*-acetyl-α-D-glucopyranosyl)-4-hetaryl-1,2,3-triazoles by the Zemplén method 4](#_Toc196488103)

[1.4 General procedure III for the *O*-peracylation of the 1-(α-D-glucopyranosyl)-4-hetaryl-
1,2,3-triazoles 4](#_Toc196488104)

[1.5 General procedure IV for the synthesis of the half-sandwich platinum-group metal complexes of the *O*-peracylated and *O*-unprotected 1-(α-D-glucopyranosyl)-4-hetaryl-1,2,3-triazoles 4](#_Toc196488105)

[1.6 Synthesis and characterization of the new compounds 5](#_Toc196488106)

[1-(2’,3’,4’,6’-Tetra-*O*-acetyl-α-D-glucopyranosyl)-4-(pyridin-2-yl)-1,2,3-triazole (**2a**) 5](#_Toc196488107)

[1-(2’,3’,4’,6’-Tetra-*O*-acetyl-α-D-glucopyranosyl)-4-(quinolin-2-yl)-1,2,3-triazole (**2b**) 5](#_Toc196488108)

[1-(α-D-Glucopyranosyl)-4-(pyridin-2-yl)-1,2,3-triazole (**3a**) 5](#_Toc196488109)

[1-(α-D-Glucopyranosyl)-4-(quinolin-2-yl)-1,2,3-triazole (**3b**) 6](#_Toc196488110)

[1-(2’,3’,4’,6’-Tetra-*O*-benzoyl-α-D-glucopyranosyl)-4-(pyridin-2-yl)-1,2,3-triazole (**4a**) 6](#_Toc196488111)

[1-(2’,3’,4’,6’-Tetra-*O*-benzoyl-α-D-glucopyranosyl)-4-(quinolin-2-yl)-1,2,3-triazole (**4b**) 6](#_Toc196488112)

[1-(2’,3’,4’,6’-Tetra-*O*-pentanoyl-α-D-glucopyranosyl)-4-(pyridin-2-yl)-1,2,3-triazole (**5a**) 7](#_Toc196488113)

[Complex **Ru-2a** 7](#_Toc196488114)

[Complex **Ir-2a** 8](#_Toc196488115)

[Complex **Ru-2b** 8](#_Toc196488116)

[Complex **Ir-2b** 9](#_Toc196488117)

[Complex **Ru-3a** 10](#_Toc196488118)

[Complex **Ir-3a** 10](#_Toc196488119)

[Complex **Ru-4a** 10](#_Toc196488120)

[Complex **Os-4a** 11](#_Toc196488121)

[Complex **Ir-4a** 12](#_Toc196488122)

[Complex **Rh-4a** 12](#_Toc196488123)

[Complex **Ru-4b** 13](#_Toc196488124)

[Complex **Os-4b** 13](#_Toc196488125)

[Complex **Ir-4b** 14](#_Toc196488126)

[Complex **Rh-4b** 14](#_Toc196488127)

[Complex **Os-5a** 15](#_Toc196488128)

[2 Copies of the ^1^H and ^13^C NMR spectra 16](#_Toc196488129)

[3 References 38](#_Toc196488130)

# Synthetic details

## General methods

Optical rotation measurements were conducted on a Jasco P-2000 polarimeter (Jasco, Easton, MD, USA) at ambient temperature, with reported values representing the average of three parallel determinations. NMR spectra were recorded using Bruker (Karlsruhe, Germany) spectrometers: DRX360 (360/90 MHz for ^1^H/^13^C) and DRX400 (400/100 MHz for ^1^H/^13^C), and Avance II 500 (500/125 MHz for ^1^H/^13^C). Me_4_Si was applied as reference for chemical shifts of ^1^H-NMR, while the residual solvent signals were used for those of ^13^C-NMR. The proton- and carbon-signal assignments for characteristic resonances of the prepared complexes were based on COSY and HSQC correlations of some representatives of the series (**Ru-2b**, **Ir-2b**, **Os-4b**, **Rh-4b**). ESI-HRMS data was obtained by measurements on a Bruker maXis II spectrometer using positive ionization mode. TLC analyses were conducted on DC Kieselgel 60 F_254_ plates (Sigma-Aldrich), with visualization achieved under UV light or by gentle heating. For purifications carried out by column chromatography, Kieselgel 60 silica gel (particle size 0.063-0.2 mm, Molar Chemicals) as stationary phase was used. Among anhydrous solvents used, pyridine was acquired from VWR Chemicals, while the others were prepared in our laboratory following established distillation protocols: halogenated solvents (CH_2_Cl_2_ and CHCl_3_) were distilled from P_4_O_10_ and stored over 4 Å molecular sieves, and methanol was dried by distillation over magnesium turnings and iodine. 2-Ethynylpyridine (TCI Chemicals), pentanoyl chloride (Alfa Aesar), TlPF_6_ (Strem Chemicals), dichloro(η^6^-*p*-cymene)ruthenium(II) dimer (**Ru-dimer**, Strem Chemicals), dichloro(η^5^-pentamethylcyclopentadienyl)iridium(III) dimer (**Ir-dimer**, Acros Organics), and dichloro(η^5^-pentamethylcyclopentadienyl)rhodium(III) dimer (**Rh-dimer**, Alfa Aesar) were purchased from the given suppliers. 2,3,4,6-Tetra-*O*-acetyl-α-D-glucopyranosyl azide (**1**) (Zhang et al., 1999), 2-ethynylquinoline (Son et al., 2013), and dichloro(η^6^-*p*-cymene)osmium(II) dimer (Godó et al., 2012) (**Os-dimer**) were synthesized in accordance with literature methods.

## General procedure I for the synthesis of 1-(2’,3’,4’,6’-tetra-*O*-acetyl-α-D-glucopyranosyl)-4-hetaryl-1,2,3-triazoles

2,3,4,6-Tetra-*O*-acetyl-α-D-glucopyranosyl azide (Zhang et al., 1999) (**1**) was dissolved in a solvent mixture of *t*-BuOH-H_2_O (15-15 mL /1 g of azide). To this solution the corresponding 2-ethynylated heterocycle (1.4 equiv.), L-ascorbic acid (0.8 equiv.) and CuSO_4_·5H_2_O (0.2 equiv.) were added. The reaction mixture was heated at 70 °C under stirring. When the TLC (1:1 EtOAc-hexane) showed complete disappearance of the starting azide (~ 1 day), the reaction mixture was diluted with water (30 mL) and extracted with CH_2_Cl_2_ (3 × 50 mL). The organic layers were combined and washed with 5% EDTA in 1M aqueous solution of NH_4_OH (30 mL), then with water (50 mL). The separated organic phase was dried over anhydrous MgSO_4_, filtered and evaporated under diminished pressure. Purification of the residual crude product was carried out by column chromatography.

## General procedure II for the deacetylation of the 1-(2’,3’,4’,6’-tetra-*O*-acetyl-α-D-glucopyranosyl)-4-hetaryl-1,2,3-triazoles by the Zemplén method

The appropriate *O*-peracetylated 1-(2’,3’,4’,6’-tetra-*O*-acetyl-α-D-glucopyranosyl)-4-hetaryl-1,2,3-triazole (**2a,b**) was dissolved in a solvent mixture of anhydrous methanol and anhydrous chloroform (2-2 mL / 100 mg of triazole). To this solution a few drops of 1 M solution of sodium methoxide in methanol was added to adjust the pH to a range of 8-9. The reaction mixture was left to stand at ambient temperature, and the transformation was monitored by TLC (1:1 EtOAc-hexane and 7:2 CHCl_3_-MeOH). After completion of the reaction, the mixture was treated with a cation exchange resin (Amberlyst 15, in H^+^ form) for neutralization. Subsequently, the resin was removed by filtration, and the solution was concentrated under reduced pressure. The pure compound was obtained by column chromatographic purification.

## General procedure III for the *O*-peracylation of the 1-(α-D-glucopyranosyl)-4-hetaryl-1,2,3-triazoles

The corresponding 1-(α-D-glucopyranosyl)-4-hetaryl-1,2,3-triazole (**3a,b**) was dissolved in anhydrous pyridine (4 mL / 50 mg triazole). To this solution the appropriate carboxylic acid chloride (4.8 equiv.) was added under stirring. The reaction mixture was subsequently heated to 60 °C and the transformation was monitored by TLC (7:2 CHCl_3_-MeOH and 1:2 EtOAc-hexane). After two hours, the TLC indicated incompleteness of the reaction, therefore, additional portions of acid chloride (2 × 4.8 equiv. per 4 hours) were added to the mixture, and then the stirring was continued at 60 °C overnight. After that, the pyridine was removed under reduced pressure, and the residue was diluted with water (20 mL) and extracted with CHCl_3_ (2 × 20 mL). The combined organic layers were extracted with saturated aqueous solution of NaHCO_3_ (3 × 20 mL), then with water (20 mL). The organic layer was dried (MgSO_4_), filtered and the solvent was removed in vacuo. The target pure compound was obtained by column chromatographic purification of the residue.

## General procedure IV for the synthesis of the half-sandwich platinum-group metal complexes of the *O*-peracylated and *O*-unprotected 1-(α-D-glucopyranosyl)-4-hetaryl-1,2,3-triazoles

To a solution of the appropriate dimeric chloro-bridged metal complex (**Ru-dimer/Os-dimer/Ir-dimer**/**Rh-dimer**) in anhydrous CH_2_Cl_2_ (1 mL / 10 mg dimer) the corresponding 1-(α-D-glucopyranosyl)-4-hetaryl-1,2,3-triazole (2.0-2.3 equiv.) and TlPF_6_ (2 equiv.) were added. Under stirring at ambient temperature, anhydrous MeOH (1 mL / 10 mg dimer) was also added to the reaction mixture to promote the precipitation of the TlCl. The stirring was continued at the same temperature until the TLC (95:5 CHCl_3_-MeOH) indicated the total consumption of the dimer (~ 1 hour). The TlCl was removed by filtration using a syringe filter (Nylon, 25 mm, 0.22 µm), and the resulting solution was evaporated in vacuo. The pure complex from the residue was obtained by trituration in a solvent mixture, recrystallization or column chromatographic purification.

## Synthesis and characterization of the new compounds

### 1-(2’,3’,4’,6’-Tetra-*O*-acetyl-α-D-glucopyranosyl)-4-(pyridin-2-yl)-1,2,3-triazole (2a)

Obtained from azide **1** (0.50 g, 1.34 mmol) and 2-ethynylpyridine (0.19 mL, 1.88 mmol) according to general procedure I. Purification by column chromatography (1:1 → 3:2 EtOAc-hexane) resulted in 0.60 g of white amorphous solid. Percent yield: 95%. R_f_ = 0.32 (4:1 EtOAc-hexane); [α]_D_ = +129 (c 0.20, CHCl_3_). ^1^H-NMR (400 MHz, CDCl_3_) δ (ppm): 8.60 (1H, ddd, *J* = 4.8, 1.8, 0.9 Hz, Py-H-6), 8.29 (1H, s, Tria-H-5), 8.22 (1H, d, *J* = 7.9 Hz, Py-H-3), 7.83 (1H, td, *J* = 7.8, 1.8 Hz, Py-H-4), 7.28 (1H, m, Py-H-5), 6.45 (1H, d, *J* = 6.1 Hz, H-1’), 6.35 (1H, dd, *J* = 10.1, 9.2 Hz, H-3’ or H-4’), 5.39 (1H, dd, *J* = 10.1, 6.1 Hz, H-2’), 5.30 (1H, dd, *J* = 10.3, 9.2 Hz, H-3’ or H-4’), 4.41 (1H, ddd, *J* = 10.3, 4.0, 2.2 Hz, H-5’), 4.29 (1H, dd, *J* = 12.7, 4.0 Hz, H-6’a), 4.03 (1H, dd, *J* = 12.7, 2.2 Hz, H-6’b), 2.07, 2.07, 2.05, 1.89 (4 × 3H, 4 s, 4 × CH_3_); ^13^C-NMR (100 MHz, CDCl_3_) δ (ppm): 170.5, 170.2, 169.8, 169.7 (4 × C=O), 149.6 (Py-C-6), 148.0 (2) (Tria-C-4, Py-C-2), 137.2 (Py-C-4), 124.4 (Tria-C-5), 123.4, 120.5 (Py-C-3, Py-C-5), 81.8 (C-1’), 71.3, 70.5, 69.7, 68.1 (C-2’ – C-5’), 61.4 (C-6’), 20.7 (2), 20.6, 20.4 (4 × CH_3_). ESI-HRMS positive mode (m/z): calculated for C_21_H_25_N_4_O_9_^+^ [M+H]^+^ 477.1616; C_21_H_24_N_4_NaO_9_ ^+^ [M+Na]^+^ 499.1435. Found: [M+H]^+^ 477.1615; [M+Na]^+^ 499.1433.

### 1-(2’,3’,4’,6’-Tetra-*O*-acetyl-α-D-glucopyranosyl)-4-(quinolin-2-yl)-1,2,3-triazole (2b)

Obtained from azide **1** (0.85 g, 2.28 mmol) and 2-ethynylquinoline (0.49 g, 3.19 mmol) according to general procedure I. Purification by column chromatography (1:2 → 1:1 EtOAc-hexane, then 1:3 EtOAc-toluene) resulted in 0.48 g of white solid. Percent yield: 40%. R_f_ = 0.26 (1:1 EtOAc-hexane); [α]_D_ = +126 (c 0.21, CHCl_3_). ^1^H-NMR (400 MHz, CDCl_3_) δ (ppm): 8.47 (1H, s, Tria-H-5), 8.37, 8.29 (2 × 1H, 2 d, *J* = 8.5 Hz for both, Qu-H-3 and Qu-H-4), 8.06, 7.86 (2 × 1H, 2 d, *J* = 8.5 and 8.1 Hz, respectively, Qu-H-5 and Qu-H-8), 7.74 (1H, pt, *J* = 8.5, 7.9 Hz, Qu-H-6 or Qu-H-7), 7.56 (1H, pt, *J* = 8.1, 7.9 Hz, Qu-H-6 or Qu-H-7), 6.48 (1H, d, *J* = 6.0 Hz, H-1’), 6.40 (1H, pt, *J* = 9.6, 9.6 Hz, H-3’ or H-4’), 5.41 (1H, dd, *J* = 10.0, 6.0 Hz, H-2’), 5.31 (1H, pt, *J* = 9.7, 9.7 Hz, H-3’ or H-4’), 4.43 (1H, ddd, *J* = 10.5, 4.0, 2.2 Hz, H-5’), 4.30 (1H, dd, *J* = 12.7, 4.0 Hz, H-6’a), 4.04 (1H, dd, *J* = 12.7, 2.2 Hz, H-6’b), 2.08 (2), 2.06, 1.90 (4 × 3H, 4 s, 4 × CH_3_); ^13^C-NMR (100 MHz, CDCl_3_) δ (ppm): 170.6, 170.3, 169.8, 169.7 (4 × C=O), 149.8, 148.4, 148.2 (Tria-C-4, Qu-C-2, Qu-C-8a), 137.2, 130.0, 129.2, 127.9, 126.7, 118.7 (Qu-C-3 – Qu-C-8), 128.0 (Qu-C-4a), 125.2 (Tria-C-5), 81.9 (C-1’), 71.3, 70.6, 69.8, 68.2 (C-2’ – C-5’), 61.4 (C-6’), 20.8 (2), 20.7 , 20.5 (4 × CH_3_). ESI-HRMS positive mode (m/z): calculated for C_25_H_27_N_4_O_9_^+^ [M+H]^+^ 527.1773; C_25_H_26_N_4_NaO_9_^+^ [M+Na]^+^ 549.1592. Found: [M+H]^+^ 527.1775; [M+Na]^+^ 549.1594.

### 1-(α-D-Glucopyranosyl)-4-(pyridin-2-yl)-1,2,3-triazole (3a)

Obtained from compound **2a** (0.97 g, 2.04 mmol) according to general procedure II. Reaction time: 4 hrs. Purification by column chromatography (7:2 CHCl_3_-MeOH) resulted in 0.58 mg of white amorphous solid. Percent yield: 90%. R_f_ = 0.50 (3:2 CHCl_3_-MeOH); [α]_D_ = +80 (c 0.21, MeOH). ^1^H-NMR (400 MHz, CD_3_OD) δ (ppm): 8.59 (2H, broad signal, Tria-H-5, Py-H-6), 8.10 (1H, d, *J* = 7.8 Hz, Py-H-3), 7.92 (1H, td, *J* = 7.8, 1.7 Hz, Py-H-4), 7.38 (1H, m, Py-H-5), 6.29 (1H, d, *J* = 5.8 Hz, H-1’), 4.40 (1H, pt, *J* = 9.3, 9.3 Hz, H-3’ or H-4’), 4.03 (1H, dd, *J* = 9.7, 5.8 Hz, H-2’), 3.87 (1H, ddd, *J* = 10.0, 5.2, 2.3 Hz, H-5’), 3.79 (1H, dd, *J* = 12.2, 2.3 Hz, H-6’a), 3.71(1H, dd, *J* = 12.2, 5.2 Hz, H-6’b), 3.54 (1H, dd, *J* = 10.0, 9.0 Hz, H-3’ or H-4’); ^13^C-NMR (100 MHz, CD_3_OD) δ (ppm): 150.9, 147.7 (Tria-C-4, Py-C-2), 150.5 (Py-C-6), 139.0 (Py-C-4), 126.5, 124.6, 121.7 (Tria-C-5, Py-C-3, Py-C-5), 87.5 (C-1’), 77.7, 74.8, 72.3, 71.4 (C-2’ – C-5’), 62.4 (C-6’). ESI-HRMS positive mode (m/z): calculated for C_13_H_16_N_4_NaO_5_^+^ [M+Na]^+^ 331.1013. Found: [M+Na]^+^ 331.1010.

### 1-(α-D-Glucopyranosyl)-4-(quinolin-2-yl)-1,2,3-triazole (3b)

Obtained from compound **2b** (0.19 g, 0.36 mmol) according to general procedure II. Reaction time: 5 hrs. Purification by column chromatography (4:1 CHCl_3_-MeOH) yielded 0.12 g of white amorphous solid. Percent yield: 92%. R_f_ = 0.38 (4:1 CHCl_3_-MeOH); [α]_D_ = +76 (c 0.09, MeOH). ^1^H-NMR (400 MHz, CD_3_OD) δ (ppm): 8.81 (1H, s, Tria-H-5), 8.39, 8.22 (2 × 1H, 2 d, *J* = 8.6 Hz for both, Qu-H-3 and Qu-H-4), 8.05, 7.92 (2 × 1H, 2 d, *J* = 8.5 and 8.0 Hz, respectively, Qu-H-5 and Qu-H-8), 7.76 (1H, pt, *J* = 8.5, 8.0 Hz, Qu-H-6 or Qu-H-7), 7.58 (1H, pt, *J* = 8.1, 8.0 Hz, Qu-H-6 or Qu-H-7), 6.35 (1H, d, *J* = 5.8 Hz, H-1’), 4.43 (1H, pt, *J* = 9.3, 9.3 Hz, H-3’ or H-4’), 4.05 (1H, dd, *J* = 9.7, 5.8 Hz, H-2’), 3.91(1H, m, H-5’), 3.81 (1H, dd, *J* = 12.1, 2.0 Hz, H-6’a), 3.73 (1H, pt, *J* = 12.1, 5.1 Hz, H-6’b), 3.56 (1H, pt, *J* = 9.4, 9.4 Hz, H-3’ or H-4’); ^13^C-NMR (100 MHz, CD_3_OD) δ (ppm): 151.3, 149.1, 148.0 (Tria-C-4, Qu-C-2, Qu-C-8a), 138.8, 131.4, 129.3, 129.1, 128.0, 119.7 (Qu-C-3 – Qu-C-8), 129.2 (Qu-C-4a), 127.3 (Tria-C-5), 87.6 (C-1’), 77.8, 74.9, 72.4, 71.4 (C-2’ – C-5’), 62.4 (C-6’). ESI-HRMS positive mode (m/z): calculated for C_17_H_18_N_4_NaO_5_^+^ [M+Na]^+^ 381.1169. Found: [M+Na]^+^ 381.1171.

### 1-(2’,3’,4’,6’-Tetra-*O*-benzoyl-α-D-glucopyranosyl)-4-(pyridin-2-yl)-1,2,3-triazole (4a)

Obtained from compound **3a** (64.1 mg, 0.21 mmol) and benzoyl chloride (3 × 117 μL, 3 × 1.01 mmol) according to general procedure III. Purification by column chromatography (1:4 → 1:2 EtOAc-hexane) resulted in 112 mg of white solid. Percent yield: 75%. R_f_ = 0.36 (1:1 EtOAc-hexane); [α]_D_ = +98 (c 0.21, CHCl_3_)_._ ^1^H-NMR (400 MHz, CDCl_3_) δ (ppm): 8.55 (1H, ddd, *J* = 4.8, 1.8, 0.9 Hz, Py-H-6), 8.30 (1H, s, Tria-H-5), 8.22-7.22 (23H, m, Ph, Py-H-3, Py-H-4, Py-H-5), 7.09 (1H, pt, *J* = 9.8, 9.8 Hz, H-3’or H-4’), 6.71 (1H, d, *J* = 6.0 Hz, H-1’), 5.97 (1H, pt, *J* = 10.1, 9.6 Hz, H-3’or H-4’), 5.84 (1H, dd, *J* = 10.0, 6.0 Hz, H-2’), 4.80 (1H, ddd, *J* =10.3, 4.1, 2.8 Hz, H-5’), 4.59 (1H, dd, *J* = 12.6, 2.8 Hz, H-6’a), 4.44 (1H, *J* = 12.6, 4.1 Hz, H-6’b); ^13^C-NMR (100 MHz, CDCl_3_) δ (ppm):166.1, 165.9, 165.4, 165.3 (4 × C=O), 149.7 (Py-C-6), 149.6, 148.2 (Tria-C-4, Py-C-2), 137.2 (Py-C-4), 133.9, 133.7, 133.3 (2), 130.1-127.9 (Ph), 124.6 (Tria-C-5), 123.4, 120.5 (Py-C-3, Py-C-5), 82.2 (C-1’), 71.8, 70.9 (2), 68.9 (C-2’ – C-5’), 62.2 (C-6’). ESI-HRMS positive mode (m/z): calculated for C_41_H_32_N_4_NaO_9_^+^ [M+Na]^+^ 747.2061. Found: 747.2058.

### 1-(2’,3’,4’,6’-Tetra-*O*-benzoyl-α-D-glucopyranosyl)-4-(quinolin-2-yl)-1,2,3-triazole (4b)

Obtained from compound **3b** (0.11 g, 0.31 mmol) and benzoyl chloride (3 × 173 μL, 3 × 1.49 mmol) according to general procedure III. Purification by column chromatography (1:4 → 1:2 EtOAc-hexane) resulted in 0.22 g of white amorphous solid. Percent yield: 91%. R_f_ = 0.50 (1:1 EtOAc-hexane); [α]_D_ = +61 (c 0.19, CHCl_3_). ^1^H-NMR (360 MHz, CDCl_3_) δ (ppm): 8.54 (1H, s, Tria-H-5), 8.38, 8.28 (2 × 1H, 2 d, *J* = 8.5 Hz for both, Qu-H-3 and Qu-H-4), 8.13-7.11 (24H, m, Ph, Qu-H-5, Qu-H-6, Qu-H-7, Qu-H-8), 7.14 (1H, pt, *J* = 9.8, 9.8 Hz, H-3’ or H-4’), 6.76 (1H, d, *J* = 6.0 Hz, H-1’), 5.99 (1H, pt, *J* = 9.9, 9.9 Hz, H-3’ or H-4’), 5.87 (1H, dd, *J* = 10.0, 6.0 Hz, H-2’), 4.83 (1H, m, H-5’), 4.60 (1H, dd, *J* = 12.6, 2.8 Hz, H-6’a), 4.45 (1H, dd, *J* = 12.6, 4.1 Hz, H-6’b); ^13^C-NMR (90 MHz, CDCl_3_) δ (ppm): 166.2, 165.9, 165.5, 165.4 (4 × C=O), 149.8, 148.5, 148.1 (Tria-C-4, Qu-C-2, Qu-C-8a), 137.2, 133.9, 133.8, 133.7, 133.3, 130.3-127.9, 126.8, 125.5, 118.8 (Ph, Tria-C-5, Qu-C-3 – Qu-C-8, Qu-C-4a), 82.4 (C-1’), 71.9, 71.0, 70.9, 69.0 (C-2’ – C-5’), 62.3 (C-6’). ESI-HRMS positive mode (m/z): calculated for C_45_H_35_N_4_O_9_^+^ [M+H]^+^ 775.2399; C_45_H_34_N_4_O_9_Na^+^ [M+Na]^+^ 797.2218. Found: [M+H]^+^ 775.2399; [M+Na]^+^ 797.2216.

### 1-(2’,3’,4’,6’-Tetra-*O*-pentanoyl-α-D-glucopyranosyl)-4-(pyridin-2-yl)-1,2,3-triazole (5a)

Obtained from compound **3a** (50 mg, 0.162 mmol) and pentanoyl chloride (3 × 95 μL, 3 × 0.780 mmol) according to general procedure III. Purification by column chromatography (1:4 EtOAc-hexane) resulted in 59 mg of pale yellow amorphous solid. Percent yield: 56%. R_f_ = 0.45 (1:1 EtOAc-hexane); [α]_D_ = +97 (c 0.21, CHCl_3_). ^1^H-NMR (360 MHz, CDCl_3_) δ (ppm): 8.60 (1H, d, *J* = 4.3 Hz, Py-H-6), 8.25 (1H, s, Tria-H-5), 8.22 (1H, d, *J* = 7.9 Hz, Py-H-3), 7.82 (1H, td, *J* = 7.9, 1.4 Hz, Py-H-4), 7.27 (1H, dd, *J* = 7.7, 4.3 Hz, Py-H-5), 6.45 (1H, d, *J* = 6.1 Hz, H-1’), 6.37 (1H, pt, *J* = 9.7, 9.7 Hz, H-3’ or H-4’), 5.36 (1H, dd, *J* = 9.7, 6.1 Hz, H-2’), 5.32 (1H, pt, *J* = 9.7, 9.7 Hz, H-3’ or H-4’), 4.43 (1H, ddd, *J* = 10.4, 4.2, 2.1 Hz, H-5’), 4.24 (1H, dd, *J* = 12.6, 4.2 Hz, H-6’a), 4.05 (1H, dd, *J* = 12.6, 2.1 Hz, H-6’b), 2.35-2.25 (6H, m, 3 × CH_2_), 2.10, 2.09 (2 × 1H, 2 t, *J* = 7.4 for both, CH_2_), 1.62-1.52, 1.39-1.26, 1.16-1.05 (16H, m, 8 × CH_2_), 0.94-0.86 (9H, m, 3 × CH_3_), 0.72 (3H, t, *J* = 7.3 Hz, CH_3_); ^13^C-NMR (90 MHz, CDCl_3_) δ (ppm): 173.4, 173.1, 172.4, 172.3 (4 × C=O), 149.7, 148.0 (Tria-C-4, Py-C-2), 149.7 (Py-C-6), 137.1 (Py-C-4), 124.4 (Tria-C-5), 123.4, 120.4 (Py-C-3, Py-C-5), 81.9 (C-1’), 71.5, 70.2, 69.9, 67.8 (C-2’ ‒ C-5’), 61.2 (C-6’), 33.9, 33.8, 33.7, 33.5 (4 × CH_2_), 27.0, 26.9, 26.8, 26.8 (4 × CH_2_), 22.3 (3), 22.1 (4 × CH_2_), 13.7 (3), 13.5 (4 × CH_3_). ESI-HRMS positive mode (m/z): calculated for C_33_H_49_N_4_O_9_^+^ [M+H]^+^ 645.3494; C_33_H_48_N_4_O_9_Na^+^ [M+Na]^+^ 667.3313. Found: [M+H]^+^ 645.3496; [M+Na]^+^ 667.3313.

### Complex Ru-2a

Obtained from the chloro-bridged **Ru-dimer** (10.0 mg, 0.016 mmol), ligand **2a** (16.3 mg, 0.034 mmol, 2.1 eq.) and TlPF_6_ (11.4 mg, 0.033 mmol) according to general procedure IV. After removal of the precipitated TlCl by filtration and evaporation of the filtrate the residual crude was dissolved in chloroform (1 mL), and diisopropyl ether (4 mL) was added. The precipitation was filtered off, washed with a 1:4 solvent mixture of chloroform-diisopropyl ether (2 × 2 mL) and dried to result in 24.8 mg of **Ru-2a** as a yellow solid. Percent yield: 81%; diastereomeric ratio: 6:5. R_f_ = 0.23 (95:5 CHCl_3_-MeOH). ^1^H-NMR (400 MHz, CDCl_3_) δ (ppm ): 9.23 (d, *J* = 5.6 Hz, major Py-H-6), 9.20 (d, *J* = 5.7 Hz, minor Py-H-6), 8.74 (s, major Tria-H-5), 8.64 (s, minor Tria-H-5), 7.98-7.86 (m, minor and major Py-H-3, Py-H-4), 7.54-7.47 (m, minor and major Py-H-5), 6.55 (pt, *J* = 10.1, 9.7 Hz, major H-3’ or H-4’), 6.53 (d, *J* = 5.6 Hz, minor H-1’), 6.45 (d, *J* = 5.7 Hz, major H-1’), 6.23 (pt, *J* = 9.1, 9.1 Hz, minor H-3’ or H-4’), 6.08, 5.95, 5.72, 5.66 (4 d, *J* = 6.1 Hz in each, minor 4 × *p*-cym-CH_Ar_), 6.05, 5.93, 5.83, 5.73 (4 d, *J* = 6.1 Hz in each, major 4 × *p*-cym-CH_Ar_), 5.64 (dd, *J* = 10.5, 5.7 Hz, major H-2’), 5.50 (dd, *J* = 9.5, 5.6 Hz, minor H-2’), 5.38 (pt, *J* = 9.8, 9.8 Hz, major H-3’ or H-4’), 5.26 (dd, *J* = 10.2, 8.8 Hz, minor H-3’ or H-4’), 4.41-4.01 (m, minor and major H-5’, H-6’a,b), 2.98 (hept, *J* = 6.9 Hz, minor *i*-Pr-C*H*), 2.88 (hept, *J* = 6.9 Hz, major *i*-Pr-C*H*), 2.18 (2), 2.13, 2.11, 2.10 (3), 2.08, 1.92, 1.87 (singlets, minor and major 4 × COCH_3_, C_6_H_4_-C*H*_3_), 1.34, 1.31 (2 d, *J* = 6.9 Hz, for both, minor 2 × *i*-Pr-C*H*_3_), 1.28, 1.17 (2 d, *J* = 6.9 Hz, for both, major 2 × *i*-Pr-C*H*_3_); ^13^C-NMR (100 MHz, CDCl_3_) δ (ppm): 170.8, 170.6, 170.5, 169.9 (2), 169.8, 169.6, 169.3 (minor and major 4 × C=O), 155.3 (major Py-C-6), 154.7 (minor Py-C-6), 148.2, 147.0 (minor Tria-C-4, Py-C-2), 147.6, 146.5 (major Tria-C-4, Py-C-2), 140.2 (major Py-C-4), 140.0 (minor Py-C-4), 127.4 (major Tria-C-5), 127.0, 123.3 (major Py-C-3, Py-C-5), 126.7, 123.0 (minor Py-C-3, Py-C-5), 126.1 (minor Tria-C-5), 106.1, 102.5 (major 2 × *p*-cym-C_qAr_), 104.7, 101.3 (minor 2 × *p*-cym-C_qAr_), 89.4, 87.1, 85.3, 84.6, 84.5, 84.4, 84.1 (2), 83.4, 83.3 (minor and major C-1’, 4 × *p*-cym-CH_Ar_), 72.4, 71.4, 70.5, 69.7, 68.7, 68.3, 68.2, 67.3 (minor and major C-2’ – C-5’), 61.2 (minor C-6’), 61.0 (major C-6’), 31.2 (minor *i*-Pr-*C*H), 31.1 (major *i*-Pr-*C*H), 23.3, 21.0 (minor 2 × *i*-Pr-*C*H_3_), 22.4, 21.8 (major 2 × *i*-Pr-*C*H_3_), 21.0, 20.9, 20.8 (2), 20.7 (2), 20.4, 20.1, 18.3, 18.1 (minor and major 4 × CO*C*H_3_, C_6_H_4_-*C*H_3_). ESI-HRMS positive mode (m/z): calculated for C_31_H_38_ClN_4_O_9_Ru^+^ [M-PF_6_]^+^ 747.1371. Found: 747.1370.

### Complex Ir-2a

Obtained from the chloro-bridged **Ir-dimer** (20.0 mg, 0.025 mmol), ligand **2a** (25.1 mg, 0.053 mmol, 2.1 eq.) and TlPF_6_ (17.6 mg, 0.050 mmol) according to general procedure IV. After removal of the precipitated TlCl by filtration and evaporation of the filtrate the residual crude was dissolved in chloroform (2 mL), and diisopropyl ether (8 mL) was added. The precipitation was filtered off, washed with a 1:3 solvent mixture of chloroform-diisopropyl ether (2 × 1 mL) and dried to result in 45 mg of **Ir-2a** as a yellow powder. Percent yield: 87%; diastereomeric ratio: 5:1. R_f_ = 0.24 (95:5 CHCl_3_-MeOH). ^1^H-NMR (400 MHz, CDCl_3_) δ (ppm): 8.84 (s, major Tria-H-5), 8.75 (s, minor Tria-H-5), 8.74 (d, *J* = 5.4 Hz, minor and major Py-H-6), 8.14-8.01 (m, minor and major Py-H-3, Py-H-4), 7.64-7.56 (m, minor and major Py-H-5), 6.55 (d, *J* = 5.7 Hz, minor H-1’), 6.53 (d, *J* = 6.0 Hz, major H-1’), 6.28 (pt, *J* = 9.8, 9.7 Hz, major H-3’ or H-4’), 6.25 (pt, *J* = 9.4, 9.2 Hz, minor H-3’ or H-4’), 5.64 (dd, *J* = 10.2, 6.0 Hz, major H-2’), 5.52 (dd, *J* = 9.7, 5.7 Hz, minor H-2’), 5.37 (pt, *J* = 9.9, 9.8 Hz, major H-3’ or H-4’), 5.33 (dd, *J* = 10.0, 8.8 Hz, minor H-3’ or H-4’), 4.35 (dd, *J* = 13.0, 3.7 Hz, minor H-6’a), 4.29 (dd, *J* = 12.8, 3.8 Hz, major H-6’a), 4.20-4.01 (m, minor and major H-5’, H-6’b), 2.09, 2.08 (4), 2.07, 1.94, 1.90 (singlets, minor and major 4 × COCH_3_), 1.82 (s, minor Cp*-CH_3_), 1.81 (s, major Cp*-CH_3_); ^13^C-NMR (100 MHz, CDCl_3_) δ (ppm): 170.7, 170.1, 169.9, 169.5 (minor 4 × C=O), 170.6, 170.2, 169.9, 169.3 (major 4 × C=O), 151.0 (major Py-C-6), 150.8 (minor Py-C-6), 148.5, 148.4 (minor Tria-C-4, Py-C-2), 148.2, 148.1 (major Tria-C-4, Py-C-2), 140.6 (major Py-C-4), 140.4 (minor Py-C-4), 128.1, 127.6, 127.3, 123.6, 123.4 (minor and major Tria-C-5, Py-C-3, Py-C-5), 89.7 (major Cp*), 89.6 (minor Cp*), 84.5 (major C-1’), 84.2 (minor C-1’), 72.0, 70.5, 68.2, 67.5 (major C-2’ – C-5’), 71.8, 70.7, 68.4, 67.9 (minor C-2’ – C-5’), 61.2 (minor C-6’), 61.1 (major C-6’), 20.8, 20.7, 20.6, 20.4, 20.1 (minor and major 4 × CO*C*H_3_), 8.8 (minor Cp*-*C*H_3_), 8.7 (major Cp*-*C*H_3_). ESI-HRMS positive mode (m/z): calculated for C_31_H_39_ClN_4_O_9_Ir^+^ [M-PF_6_]^+^ 839.2023. Found: 839.2020.

### Complex Ru-2b

Obtained from the chloro-bridged **Ru-dimer** (10 mg, 0.016 mmol), ligand **2b** (19.8 mg, 0.038 mmol, 2.3 eq.) and TlPF_6_ (11.4 mg, 0.033 mmol) according to general procedure IV. After removal of the precipitated TlCl by filtration and evaporation of the filtrate the residual crude was dissolved in chloroform (2 mL), and diisopropyl ether (8 mL) was added. The precipitation was filtered off, washed with a 1:3 solvent mixture of chloroform-diisopropyl ether (2 × 1 mL) and dried to result in 34.5 mg of **Ru-2b** as a yellowish-brown powder. Percent yield: 96%; diastereomeric ratio: 7:1. R_f_ = 0.25 (95:5 CHCl_3_-MeOH). ^1^H-NMR (400 MHz, CDCl_3_) δ (ppm): 9.03 (s, major Tria-H-5), 8.90 (s, minor Tria-H-5), 8.83 (d, *J* = 8.8 Hz, minor Qu-H-8), 8.79 (d, *J* = 8.7 Hz, major Qu-H-8), 8.46 (d, *J* = 7.6 Hz, major Qu-H-4), 8.42 (d, *J* = 8.2 Hz, minor Qu-H-4), 8.13 (d, *J* = 7.6 Hz, major Qu-H-3), 8.05 (d, *J* = 8.2 Hz, minor Qu-H-3), 8.14-7.72 (m, minor and major Qu-H-5, Qu-H-7), 7.77 (t, *J* = 7.4 Hz, major Qu-H-6), 7.74 (t, *J* = 7.5 Hz, minor Qu-H-6), 6.59 (d, *J* = 4.9 Hz, minor H-1’), 6.58 (pt, *J* = 10.0, 9.9 Hz, major H-3’), 6.53 (d, *J* = 5.2 Hz, major H-1’), 6.13 (pt, *J* = 8.7, 8.5 Hz, minor H-3’), 6.10, 6.09, 5.85, 5.71 (4 d, *J* = 5.9 Hz in each, minor 4 × *p*-cym-CH_Ar_), 6.09, 6.02, 5.81, 5.65 (4 d, *J* = 5.9 Hz in each, major 4 × *p*-cym-CH_Ar_), 5.72 (dd, *J* = 10.5, 5.2 Hz, major H-2’), 5.57 (dd, *J* = 9.2, 4.9 Hz, minor H-2’), 5.42 (pt, *J* = 9.9, 9.9 Hz, major H-4’), 5.28 (dd, *J* = 10.2, 8.3 Hz, minor H-4’), 4.56-4.02 (m, minor and major H-5’, H-6’a,b), 2.95 (hept, *J* = 6.9 Hz, minor *i*-Pr-C*H*), 2.79 (hept, *J* = 6.9 Hz, major *i*-Pr-C*H*), 2.13, 2.12, 2.08, 2.05, 2.03 (singlets, major 4 × COC*H*_3_, C_6_H_4_-C*H*_3_), 2.12 (3), 1.90, 1.86 (singlets, minor 4 × COC*H*_3_, C_6_H_4_-C*H*_3_), 1.33, 1.25 (2 d, *J* = 6.9 Hz for both, minor 2 × *i*-Pr-C*H*_3_), 1.20 (2) (d, *J* = 6.9 Hz, major 2 × *i*-Pr-C*H*_3_); ^13^C-NMR (100 MHz, CDCl_3_) δ (ppm): 170.8, 170.7, 169.9, 169.8 (minor 4 × C=O), 170.7, 170.6, 169.7, 169.3 (major 4 × C=O), 150.0, 148.2, 147.8 (minor Tria-C-4, Qu-C-2, Qu-C-8a), 149.6, 148.3, 147.7 (major Tria-C-4, Qu-C-2, Qu-C-8a), 141.2 (major Qu-C-4), 140.9 (minor Qu-C-4), 132.7 (major Qu-C-5), 132.4 (minor Qu-C-5), 129.6 (major Tria-C-5), 129.5 (major Qu-C-7), 129.4 (minor Qu-C-7), 129.2 (major and minor Qu-C-4a), 129.1 (major Qu-C-6, Qu-C-8), 128.9 (minor Qu-C-6, Qu-C-8), 128.2 (minor Tria-C-5), 119.9 (major Qu-C-3), 119.3 (minor Qu-C-3), 105.0, 103.3 (major 2 × *p*-cym-C_qAr_), 104.6, 101.4 (minor 2 × *p*-cym-C_qAr_), 89.4, 86.3, 84.2, 84.1 (minor 4 × *p*-cym-CH_Ar_), 89.4, 84.5, 84.4, 84.2 (major 4 × *p*-cym-CH_Ar_), 84.9 (major C-1’), 84.8 (minor C-1’), 72.4 (major C-5’), 71.8 (minor C-5’), 70.6 (minor C-3’), 69.9 (major C-3’), 68.6 (minor C-2’), 68.2 (2) (minor C-4’, major C-2’), 67.3 (major C-4’), 61.3 (minor C-6’), 61.1 (major C-6’), 31.3 (minor *i*-Pr-*C*H), 31.2 (major *i*-Pr-*C*H), 23.2, 21.1 (major 2 × *i*-Pr-*C*H_3_), 23.1, 21.3 (minor 2 × *i*-Pr-*C*H_3_), 20.9, 20.8, 20.7, 20.6, 20.4 (minor and major 4 × CO*C*H_3_), 18.4 (major C_6_H_4_-*C*H_3_), 18.2 (minor C_6_H_4_-*C*H_3_). ESI-HRMS positive mode (m/z): calculated for C_35_H_40_ClN_4_O_9_Ru^+^ [M-PF_6_]^+^ 797.1528. Found: 797.1528.

### Complex Ir-2b

Obtained from the chloro-bridged **Ir-dimer** (10 mg, 0.013 mmol), ligand **2b** (15.2 mg, 0.029 mmol, 2.3 eq.) and TlPF_6_ (8.8 mg, 0.025 mmol) according to general procedure IV. After removal of the precipitated TlCl by filtration and evaporation of the filtrate the residual crude was dissolved in chloroform (2 mL), and diisopropyl ether (8 mL) was added. The precipitation was filtered off, washed with a 1:3 solvent mixture of chloroform-diisopropyl ether (2 × 1 mL) and dried to result in 26 mg of **Ir-2b** as a yellow powder. Percent yield: 87%; diastereomeric ratio: 4:1. R_f_ = 0.31 (95:5 CHCl_3_-MeOH). ^1^H-NMR (400 MHz, CDCl_3_) δ (ppm): 9.01 (s, major Tria-H-5), 8.98 (s, minor Tria-H-5), 8.51 (d, *J* = 8.5 Hz, major Qu-H-4), 8.49 (d, *J* = 8.5 Hz, major Qu-H-8), 8.48 (d, *J* = 8.7 Hz minor Qu-H-4), 8.41 (d, *J* = 8.6 Hz, minor Qu-H-8), 8.23 (d, *J* = 8.5 Hz major Qu-H-3), 8.20 (d, *J* = 8.7 Hz, minor Qu-H-3), 8.02 (dd, *J* = 8.2, 1.4 Hz major Qu-H-5), 7.99 (dd, *J* = 8.3, 1.4 Hz, minor Qu-H-5), 7.93 (ddd, *J* = 8.5, 6.9, 1.4 Hz major Qu-H-7), 7.90 (ddd, *J* = 8.6, 6.9, 1.4 Hz, minor Qu-H-7), 7.78 (ddd, *J* = 8.2, 6.9, 1.1 Hz major Qu-H-6), 7.75 (ddd, *J* = 8.3, 6.9, 1.1 Hz, minor Qu-H-6), 6.65 (d, *J* = 5.4 Hz, minor H-1’), 6.57 (d, *J* = 6.1 Hz, major H-1’), 6.25 (dd, *J* = 10.2, 9.4 Hz, major H-3’), 6.16 (dd, *J* = 9.1, 8.1 Hz, minor H-3’), 5.75 (dd, *J* = 10.2, 6.1 Hz, major H-2’), 5.57 (dd, *J* = 9.1, 5.4 Hz, minor H-2’), 5.38 (dd, *J* = 10.3, 9.4 Hz, major H-4’), 5.32 (dd, *J* = 10.2, 8.1 Hz, minor H-4’), 4.44 (dd, *J* = 13.0, 4.1 Hz, minor H-6’a), 4.32-4.23 (m, minor H-5’, H-6’b), 4.29 (dd, *J* = 12.8, 3.8 Hz, major H-6’a), 4.18 (ddd, *J* = 10.3, 3.8, 2.1 Hz, major H-5’), 4.01 (dd, *J* = 12.8, 2.1 Hz, major H-6’b), 2.11, 2.10, 2.09, 1.93 (4 s, minor 4 × COC*H*_3_), 2.10, 2.08, 2.07, 2.00 (4 s, major 4 × COC*H*_3_), 1.73 (s, minor Cp*-CH_3_), 1.70 (s, major Cp*-CH_3_); ^13^C-NMR (100 MHz, CDCl_3_) δ (ppm): 170.7, 170.0, 169.9, 169.6 (minor 4 × C=O), 170.6, 170.3, 169.7, 169.4 (major 4 × C=O), 149.7, 149.6, 145.6 (minor Tria-C-4, Qu-C-2, Qu-C-8a), 149.5 (2), 145.8 (major Tria-C-4, Qu-C-2, Qu-C-8a), 141.7 (major Qu-C-4), 141.5 (minor Qu-C-4), 132.5 (major Qu-C-7), 132.3 (minor Qu-C-7), 130.2 (major Tria-C-5), 130.1 (minor Qu-C-8), 129.9, (major Qu-C-8), 129.7 (major Qu-C-4a), 129.6 (minor Qu-C-4a), 129.5 (major Qu-C-5), 129.4 (minor Qu-C-5), 129.2 (major Qu-C-6), 129.1 (minor Qu-C-6), 128.6 (minor Tria-C-5), 119.5 (major Qu-C-3), 119.4 (minor Qu-C-3), 90.2 (2) (minor and major Cp*), 84.9 (major C-1’), 84.6 (minor C-1’), 72.2 (minor C-5’), 72.0 (major C-5’), 70.7 (major C-3’), 70.6 (minor C-3’), 68.3 (minor C-2’), 67.9 (major C-2’), 67.8 (minor C-4’), 67.5 (major C-4’), 61.3 (minor C-6’), 61.0 (major C-6’), 20.8, 20.7, 20.6, 20.4, 20.1 (minor and major 4 × CO*C*H_3_), 9.3 (minor Cp*-*C*H_3_), 9.2 (major Cp*-*C*H_3_). ESI-HRMS positive mode (m/z): calculated for C_35_H_41_ClN_4_O_9_Ir^+^ [M-PF_6_]^+^ 889.2179. Found: 889.2179.

### Complex Ru-3a

Obtained from the chloro-bridged **Ru-dimer** (20.0 mg, 0.033 mmol), ligand **3a** (20.1 mg, 0.065 mmol, 2.0 eq.), and TlPF_6_ (22.8 mg, 0.065 mmol) according to general procedure IV. After removal of the precipitated TlCl by filtration and evaporation of the filtrate the crude product was recrystallized from a solvent mixture of isopropyl alcohol and methanol (3:0.5 mL). After filtration and washing the precipitation with cold isopropyl alcohol (1 mL) 28 mg of **Ru-3a** was isolated as a greenish-brown powder. Percent yield: 44%; diastereomeric ratio: 1:1. ^1^H-NMR (400 MHz, CD_3_OD) δ (ppm): 9.40 (2H, d, *J* = 5.6 Hz, 2 × Py-H-6), 9.09, 9.07 (2 × 1H, 2 s, 2 × Tria-H-5), 8.19-8.11 (4H, m, 2 × Py-H-3, 2 × Py-H-4), 7.68-7.64 (2H, m, 2 × Py-H-5), 6.42, 6.39 (2 × 1H, 2 d, *J* = 5.8 Hz for both, 2 × H-1’), 6.15 (2), 6.07, 6.02, 5.93, 5.91, 5.85, 5.81 (8 × 1H, 8 d, *J* = 6.0 Hz in each, 2 × 4 × *p*-cym-CH_Ar_), 4.33 (1H, pt, *J* = 9.3, 9.2 Hz, H-3’ or H-4’), 4.25 (1H, dd, *J* = 9.7, 8.7 Hz, H-3’ or H-4’), 4.10, 4.06 (2 × 1H, 2 dd, *J* = 9.6, 5.8 Hz for both, 2 × H-2’), 4.05, 3.96 (2 × 1H, 2 ddd, *J* = 9.9, 5.5, 2.2 Hz and 10.0, 4.9, 2.4 Hz, respectively, 2 × H-5’), 3.89, 3.82 (2 × 1H, 2 dd, *J* = 12.1, 2.2 Hz and 12.2, 2.4 Hz, respectively, 2 × H-6’a), 3.77, 3.75 (2 × 1H, 2 dd, *J* = 12.1, 5.5 Hz and 12.2, 4.9 Hz, respectively, 2 × H-6’b), 3.63 (1H, dd, *J* = 9.9, 9.0 Hz, H-3’ or H-4’), 3.59 (1H, dd, *J* = 10.1, 9.1 Hz, H-3’ or H-4’), 2.78, 2.75 (2 × 1H, 2 hept, *J* = 6.9 Hz for both, 2 × *i*-Pr-C*H*), 2.24, 2.23 (2 × 3H, 2 s, 2 × C_6_H_4_-C*H*_3_), 1.19, 1.17, 1.10, 1.06 (2 × 2 × 3H, 4 d, *J* = 6.9 Hz in each, 2 × 2 × *i*-Pr-C*H*_3_); ^13^C-NMR (100 MHz, CD_3_OD) δ (ppm): 156.8, 156.7 (2 × Py-C-6), 149.6, 149.5, 147.3, 147.0 (2 × Tria-C-4, 2 × Py-C-2), 141.4 (2) (2 × Py-C-4), 128.2 (2) (2 × Tria-C-5), 127.6, 127.5, 123.7, 123.6 (2 × Py-C-3, 2 × Py-C-5), 106.6, 106.4, 104.0, 103.9 (2 × 2 × *p*-cym-C_qAr_), 90.2, 90.0 (2 × C-1’), 87.8, 87.7, 86.0, 85.8, 85.5, 85.3, 84.9, 84.8 (2 × 4 × *p*-cym-CH_Ar_), 79.2, 78.6, 74.8, 74.4, 71.9, 71.8, 71.0, 70.8 (2 × C-2’ – C-5’), 62.4, 62.1 (2 × C-6’), 32.4, 32.3 (2 × *i*-Pr-*C*H), 22.9, 22.7, 21.8, 21.7 (2 × 2 × *i*-Pr-*C*H_3_), 18.8 (2) (2 × C_6_H_4_-*C*H_3_). ESI-HRMS positive mode (m/z): calculated for C_23_H_30_ClN_4_O_5_Ru^+^ [M-PF_6_]^+^ 579.0946. Found: 579.0946.

### Complex Ir-3a

Obtained from the chloro-bridged **Ir-dimer** (20.0 mg, 0.025 mmol), ligand **3a** (15.5 mg, 0.050 mmol, 2.0 eq.) and TlPF_6_ (17.5 mg, 0.050 mmol) according to general procedure IV. After removal of the precipitated TlCl by filtration and evaporation of the filtrate the crude product was recrystallized from a solvent mixture of isopropyl alcohol and methanol (3:0.5 mL)*.* After filtration and washing the precipitation with cold isopropyl alcohol (1 mL) 21.0 mg of **Ir-3a** was isolated as a yellow powder. Percent yield: 51%; diastereomeric ratio: 1:1. ^1^H-NMR (400 MHz, CD_3_OD) δ (ppm): 9.15, 9.14 (2 × 1H, 2 s, 2 × Tria-H-5), 8.93 (2H, d, *J* = 5.7 Hz, 2 × Py-H-6), 8.28-8.19 (4H, m, 2 × Py-H-3, 2 × Py-H-4), 7.73-7.70 (2H, m, 2 × Py-H-5), 6.45, 6.43 (2 × 1H, 2 d, *J* = 5.9 Hz and 5.7 Hz, respectively, 2 × H-1’), 4.28-3.55 (12H, m, 2 × H-2’ – H-5’, 2 × H-6’a,b), 1.79 (30H, 1 signal, minor 2 × 5 × Cp*-CH_3_); ^13^C-NMR (100 MHz, CD_3_OD) δ (ppm): 153.4, 153.3 (2 × Py-C-6), 149.8, 149.7, 148.5, 148.3 (2 × Tria-C-4, 2 × Py-C-2), 141.9 (2) (2 × Py-C-4), 128.6, 123.6, 123.5 (2 × Tria-C-5, 2 × Py-C-3, 2 × Py-C-5), 90.9 (2) (2 × Cp*), 90.3, 90.2 (2 × C-1’), 79.4, 78.7, 75.0, 74.6, 71.9, 71.8, 71.0, 70.8 (2 × C-2’ – C-5’), 62.5, 62.2 (2 × C-6’), 8.9 (2) (2 × Cp*-*C*H_3_). ESI-HRMS positive mode (m/z): calculated for C_23_H_31_ClN_4_O_5_Ir^+^ [M-PF_6_]^+^ 671.1599. Found: 671.1596.

### Complex Ru-4a

Obtained from the chloro-bridged **Ru-dimer** (10.0 mg, 0.016 mmol), ligand **4a** (24.9 mg, 0.034 mmol, 2.1 eq.) and TlPF_6_ (11.4 mg, 0.033 mmol) according to general procedure IV. After removal of the precipitated TlCl by filtration and evaporation of the filtrate the residual crude was dissolved in chloroform (2 mL), and diisopropyl ether (8 mL) was added. The precipitation was filtered off, washed with a 1:4 solvent mixture of chloroform-diisopropyl ether (2 × 2 mL) and dried to result in 44.8 mg of **Ru-4a** as an orange powder. Percent yield: 91%; diastereomeric ratio: 2:1. R_f_ = 0.28 (95:5 CHCl_3_-MeOH). ^1^H-NMR (400 MHz, CDCl_3_) δ (ppm): 9.32 (d, *J* = 5.6 Hz, major Py-H-6), 9.23 (d, *J* = 5.6 Hz, minor Py-H-6), 9.07 (s, major Tria-H-5), 8.94 (s, minor Tria-H-5), 8.06-7.23 (m, minor and major Ph, Py-H-3 – Py-H-5), 7.16 (pt, *J* = 10.1, 10.0 Hz, major H-3’ or H-4’), 6.99 (d, *J* = 5.6 Hz, major H-1’), 6.92 (pt, *J* = 9.1, 9.1 Hz, minor H-3’ or H-4’), 6.72 (d, *J* = 5.6 Hz, minor H-1’), 6.22-5.76 (m, minor and major 4 × *p*-cym-CH_Ar_, H-2’, H-3’ or H-4’), 4.84-4.47 (m, minor and major H-5’, H-6’a,b), 3.01 (hept, *J* = 6.9 Hz, minor *i*-Pr-C*H*), 2.84 (hept, *J* = 6.9 Hz, major *i*-Pr-C*H*), 2.22 (s, minor C_6_H_4_-C*H*_3_), 2.15 (s, major C_6_H_4_-C*H*_3_), 1.31, 1.27 (2 d, *J* = 6.9 Hz for both, minor 2 × *i*-Pr-C*H*_3_), 1.20, 1.06 (2 d, *J* = 6.9 Hz for both, major 2 × *i*-Pr-C*H*_3_); ^13^C-NMR (100 MHz, CDCl_3_) δ (ppm): 166.4, 166.1, 165.6, 165.1 (major 4 × C=O), 166.3, 165.5, 165.3, 165.0 (minor 4 × C=O), 155.8 (major Py-C-6), 154.9 (minor Py-C-6), 147.9, 147.0 (minor Tria-C-4, Py-C-2), 147.1, 146.7 (major Tria-C-4, Py-C-2), 140.2 (major Py-C-4), 139.9 (minor Py-C-4), 134.2, 134.0, 133.9, 133.7, 133.6, 133.5, 133.4, 130.3-128.6, 128.0, 127.6, 127.3, 126.7, 123.4, 123.0 (minor and major Ph, Tria-C-5, Py-C-3, Py-C-5), 105.8, 102.6 (major 2 × *p*-cym-C_qAr_), 105.0, 101.8 (minor 2 × *p*-cym-C_qAr_), 88.7 (minor C-1’), 87.7 (major C-1’), 85.0 (2), 84.8 (2), 84.1, 84.0, 83.8, 83.6 (minor and major 4 × *p*-cym-CH_Ar_), 73.0, 70.6, 70.1, 68.0 (major C-2’ – C-5’), 72.1, 70.9, 69.2, 68.4 (minor C-2’ – C-5’), 62.2 (minor C-6’), 61.8 (major C-6’), 31.2 (2) (minor and major *i*-Pr-*C*H), 23.0, 21.6 (minor 2 × *i*-Pr-*C*H_3_), 22.7, 21.3 (major 2 × *i*-Pr-*C*H_3_), 18.6 (major C_6_H_4_-*C*H_3_), 18.3 (minor C_6_H_4_-*C*H_3_). ESI-HRMS positive mode (m/z): calculated for C_51_H_46_ClN_4_O_9_Ru^+^ [M-PF_6_]^+^ 995.2002. Found: 995.2003.

### Complex Os-4a

Obtained from the chloro-bridged **Os-dimer** (10.0 mg, 0.013 mmol), ligand **4a** (21.1 mg, 0.029 mmol, 2.3 eq.) and TlPF_6_ (8.84 mg, 0.025 mmol) according to general procedure IV. After removal of the precipitated TlCl by filtration and evaporation of the filtrate the residual crude was dissolved in chloroform (1 mL), and diisopropyl ether (4 mL) was added. The precipitation was filtered off, washed with a 1:3 solvent mixture of chloroform-diisopropyl ether (2 × 1 mL) and dried to result in 31 mg of **Os-4a** as a yellowish-brown powder. Percent yield: 87%; diastereomeric ratio: 4:1. R_f_ = 0.30 (95:5 CHCl_3_-MeOH). ^1^H-NMR (400 MHz, CDCl_3_) δ (ppm): 9.28 (d, *J* = 5.5 Hz, major Py-H-6), 9.18 (d, *J* = 5.8 Hz, minor Py-H-6), 9.14 (s, major Tria-H-5), 9.01 (s, minor Tria-H-5), 8.13-7.22 (m, minor and major Ph, Py-H-3 – Py-H-5), 7.09 (pt, *J* = 9.9, 9.9 Hz, major H-3’ or H-4’), 7.01 (d, *J* = 5.6 Hz, major H-1’), 6.83 (pt, *J* = 8.9, 8.8 Hz, minor H-3’ or H-4’), 6.77 (d, *J* = 5.6 Hz, minor H-1’), 6.34-5.83 (minor and major 4 × *p*-cym-CH_Ar_, H-2’, H-3’ or H-4’), 4.84-4.48 (m, minor and major H-5’, H-6’a,b), 2.82 (hept, *J* = 6.9 Hz, minor *i*-Pr-C*H*), 2.68 (hept, *J* = 6.9 Hz, major *i*-Pr-C*H*), 2.29 (s, minor C_6_H_4_-C*H*_3_), 2.23 (s, major C_6_H_4_-C*H*_3_), 1.23, 1.21 (2 d, *J* = 6.9 Hz for both, minor 2 × *i*-Pr-C*H*_3_), 1.17, 1.00 (2 d, *J* = 6.9 Hz for both, major 2 × *i*-Pr-C*H*_3_); ^13^C-NMR (100 MHz, CDCl_3_) δ (ppm): 166.4, 166.1, 165.5, 165.1 (major 4 × C=O), 166.3, 165.4, 165.3, 165.0 (minor 4 × C=O), 155.8 (major Py-C-6), 155.1 (minor Py-C-6), 148.7, 148.4 (minor Tria-C-4, Py-C-2), 148.0, 147.9 (major Tria-C-4, Py-C-2), 140.4 (major Py-C-4), 140.2 (minor Py-C-4), 134.2, 134.1, 133.9, 133.8, 133.7, 133.6, 133.5, 133.4, 130.3-128.6, 127.9, 127.8, 127.6, 127.2, 123.2, 122.9 (minor and major Ph, Tria-C-5, Py-C-3, Py-C-5), 96.4, 94.8 (major 2 × *p*-cym-C_qAr_), 95.5, 93.7 (minor 2 × *p*-cym-C_qAr_), 84.9 (2) (minor and major C-1’), 80.3, 77.0, 75.0, 74.9, 72.3, 70.7, 69.2, 68.3 (minor 4 × *p*-cym-CH_Ar_, C-2’ – C-5’), 79.7, 76.7, 75.3, 74.4, 73.1, 70.4, 70.1, 68.0 (major 4 × *p*-cym-CH_Ar_, C-2’ – C-5’), 62.2 (minor C-6’), 61.8 (major C-6’), 31.3 (minor *i*-Pr-*C*H), 31.2 (major *i*-Pr-*C*H), 23.2, 21.8 (minor 2 × *i*-Pr-*C*H_3_), 23.0, 21.6 (major 2 × *i*-Pr-*C*H_3_), 18.6 (major C_6_H_4_-*C*H_3_), 18.3 (minor C_6_H_4_-*C*H_3_). ESI-HRMS positive mode (m/z): calculated for C_51_H_46_ClN_4_O_9_Os^+^ [M-PF_6_]^+^ 1085.2556. Found: 1085.2553.

### Complex Ir-4a

Obtained from the chloro-bridged **Ir-dimer** (20.0 mg, 0.025 mmol), ligand **4a** (36.4 mg, 0.050 mmol, 2.0 eq.) and TlPF_6_ (17.5 mg, 0.050 mmol) according to general procedure IV. After removal of the precipitated TlCl by filtration and evaporation of the filtrate the residual crude was dissolved in chloroform (2 mL), and diisopropyl ether (10 mL) was added. The precipitation was filtered off, washed with a 1:4 solvent mixture of chloroform-diisopropyl ether (2 × 1 mL) and dried to result in 56.5 mg of **Ir-4a** as a yellow powder. Percent yield: 91%; diastereomeric ratio: 3:2. R_f_ = 0.27 (95:5 CHCl_3_-MeOH). ^1^H-NMR (400 MHz, CDCl_3_) δ (ppm): 8.98 (s, major Tria-H-5), 8.84 (s, minor Tria-H-5), 8.76 (d, *J* = 5.7 Hz, major Py-H-6), 8.74 (d, *J* = 5.5 Hz, minor Py-H-6), 8.11-7.22 (m, minor and major Ph, Py-H-3 – Py-H-5), 6.95 (d, *J* = 5.8 Hz, major H-1’), 6.94 (pt, *J* = 9.5, 9.0 Hz, minor H-3’ or H-4’), 6.92 (pt, *J* = 9.9, 9.4 Hz, major H-3’ or H-4’), 6.70 (d, *J* = 5.8 Hz, minor H-1’), 6.18 (dd, *J* = 9.8, 5.8 Hz, minor H-2’), 6.02 (pt, *J* = 9.7, 9.5 Hz, major H-3’ or H-4’), 5.98 (dd, *J* = 10.2, 5.8 Hz, major H-2’), 5.97 (pt, *J* = 9.6, 9.2 Hz, minor H-3’ or H-4’), 4.77-4.43 (m, minor and major H-5’, H-6’a,b), 1.88 (s, minor Cp*-CH_3_), 1.85 (s, major Cp*-CH_3_); ^13^C-NMR (100 MHz, CDCl_3_) δ (ppm): 166.3, 165.6, 165.1, 165.0 (minor 4 × C=O), 166.1, 166.0, 165.4, 165.0 (major 4 × C=O), 151.4 (major Py-C-6), 150.8 (minor Py-C-6), 148.8, 148.5 (minor Tria-C-4, Py-C-2), 148.3, 147.8 (major Tria-C-4, Py-C-2), 140.7 (major Py-C-4), 140.2 (minor Py-C-4), 134.1, 134.0, 133.9, 133.7, 133.5, 133.4, 133.3, 130.4, 130.0-128.5, 128.0, 127.9, 127.7, 127.3, 123.6, 123.4 (minor and major Ph, Tria-C-5, Py-C-5, Py-C-3), 89.9 (major Cp*), 89.8 (minor Cp*), 85.0 (major C-1’), 84.9 (minor C-1’), 72.7, 70.8, 70.0, 68.1 (major C-2’ – C-5’), 71.9, 71.4, 68.7, 68.4 (minor C-2’ – C-5’), 62.2 (minor C-6’), 61.8 (major C-6’), 8.9 (2) (minor and major Cp*-*C*H_3_). ESI-HRMS positive mode (m/z): calculated for C_51_H_47_ClN_4_O_9_Ir^+^ [M-PF_6_]^+^ 1087.2651. Found: 1087.2657.

### Complex Rh-4a

Obtained from the chloro-bridged **Rh-dimer** (10.0 mg, 0.016 mmol), ligand **4a** (25.8 mg, 0.036 mmol, 2.2 eq.) and TlPF_6_ (11.3 mg, 0.032 mmol) according to general procedure IV. After removal of the precipitated TlCl by filtration and evaporation of the filtrate the residual crude was dissolved in chloroform (2 mL), and diisopropyl ether (10 mL) was added. The precipitation was filtered off, washed with a 1:4 solvent mixture of chloroform-diisopropyl ether (2 × 1 mL) and dried to result in 41.2 mg of **Rh-4a** as an orange powder. Percent yield: 91%; diastereomeric ratio: 1:1. R_f_ = 0.30 (95:5 CHCl_3_-MeOH). ^1^H-NMR (400 MHz, CDCl_3_) δ (ppm): 8.85, 8.75 (2 × 1H, 2 s, 2 × Tria-H-5), 8.74, 8.72 (2 × 1H, 2 d, *J* = 5.7 Hz for both, 2 × Py-H-6), 8.07-7.23 (46H, m, Ph, 2 × Py-H-3 – Py-H-5), 6.99, 6.97 (2 × 1H, 2 pt, *J* = 9.4, 9.4 Hz and 9.9, 9.9 Hz, respectively, 2 × H-3’ or H-4’), 6.94, 6.67 (2 × 1H, 2 d, *J* = 6.1 Hz and 5.7 Hz, respectively, 2 × H-1’), 6.16, 5.94 (2 × 1H, 2 dd, *J* = 9.8, 5.7 Hz and 10.2, 5.8 Hz, respectively, 2 × H-2’), 6.04, 5.98 (2 × 1H, 2 pt, *J* = 9.9, 9.8 Hz and 9.6, 9.5 Hz, respectively, 2 × H-3’ or H-4’), 4.77-4.42 (6H, m, 2 × H-5’, 2 × H-6’a,b), 1.89, 1.83 (2 × 15H, 2 s, 2 × 5 × Cp*-CH_3_); ^13^C-NMR (100 MHz, CDCl_3_) δ (ppm): 166.4, 166.1, 166.0, 165.7, 165.5, 165.2, 165.0 (2) (2 × 4 × C=O), 151.3, 150.7 (2 × Py-C-6), 147.5, 147.1, 146.8, 146.6 (2 × Tria-C-4, 2 × Py-C-2), 140.4, 139.9 (2 × Py-C-4), 134.2, 134.0, 133.9, 133.7, 133.5, 133.4, 133.3, 130.3-128.5, 127.7, 127.6, 126.9, 126.4, 123.5, 123.3 (Ph, 2 × Tria-C-5, 2 × Py-C-5, 2 × Py-C-3), 97.8, 97.7, 97.6 (2) (2 × Cp*) 84.7 (2) (2 × C-1’), 72.5, 71.8, 71.4, 70.7, 70.2, 68.7, 68.5, 68.1 (2 × C-2’ – C-5’), 62.2, 61.8 (2 × C-6’), 9.2, 9.1 (2 × Cp*-*C*H_3_). ESI-HRMS positive mode (m/z): calculated for C_51_H_47_ClN_4_O_9_Rh^+^ [M-PF_6_]^+^ 997.2081. Found: 997.2082.

### Complex Ru-4b

Obtained from the chloro-bridged **Ru-dimer** (10 mg, 0.016 mmol), ligand **4b** (26.6 mg, 0.034 mmol, 2.1 eq.) and TlPF_6_ (11.4 mg, 0.033 mmol) according to general procedure IV. After removal of the precipitated TlCl by filtration and evaporation of the filtrate the residual crude was dissolved in chloroform (2 mL), and diisopropyl ether (8 mL) was added. The precipitation was filtered off, washed with a 1:4 solvent mixture of chloroform-diisopropyl ether (2 × 1 mL) and dried to result in 40 mg of **Ru-4b** as a yellow powder. Percent yield: 98%; diastereomeric ratio: 5:1. R_f_ = 0.35 (95:5 CHCl_3_-MeOH). ^1^H-NMR (400 MHz, CDCl_3_) δ (ppm): 9.11 (s, major Tria-H-5), 8.96 (s, minor Tria-H-5), 8.82 (d, *J* = 8.8 Hz, minor Qu-H-8), 8.77 (d, *J* = 8.8 Hz, major Qu-H-8), 8.37 (d, *J* = 8.5 Hz, major Qu-H-4), 8.27 (d, *J* = 8.5 Hz, minor Qu-H-4), 8.10-7.24 (m, minor and major Ph, Qu-H-3, Qu-H-5 – Qu-H-7), 7.15 (pt, *J* = 9.8, 9.8 Hz, major H-3’ or H-4’), 7.01 (d, *J* = 5.6 Hz, major H-1’), 6.83 (pt, *J* = 8.6, 8.5 Hz, minor H-3’ or H-4’), 6.77 (d, *J* = 5.5 Hz, minor H-1’), 6.25 (dd, *J* = 9.0, 5.5 Hz, minor H-2’), 6.15, 6.00, 5.95, 5.55 (4 d, *J* = 5.9 Hz in each, major 4 × *p*-cym-CH_Ar_), 6.14, 6.07, 5.90, 5.71 (4 d, *J* = 5.9 Hz in each, minor 4 × *p*-cym-CH_Ar_), 6.08 (pt, *J* = 9.7, 9.7 Hz, major H-3’ or H-4’), 5.98 (dd, *J* = 10.1, 5.6 Hz, major H-2’), 5.90 (pt, *J* = 9.4, 8.1 Hz, minor H-3’ or H-4’), 5.00 (ddd, *J* = 9.9, 3.2, 2.5 Hz, minor H-5’), 4.95 (dd, *J* = 12.9, 2.5 Hz, minor H-6’a), 4.72 (ddd, *J* = 10.0, 3.7, 2.6 Hz, major H-5’), 4.66 (dd, *J* = 12.9, 3.2 Hz, minor H-6’b), 4.62 (dd, *J* = 12.8, 2.6 Hz, major H-6’a), 4.51 (dd, *J* = 12.8, 3.7 Hz, major H-6’b), 2.92 (hept, *J* = 6.9 Hz, minor *i*-Pr-C*H*), 2.68 (hept, *J* = 6.9 Hz, major *i*-Pr-C*H*), 1.95 (s, major C_6_H_4_-C*H*_3_), 1.89 (s, minor C_6_H_4_-C*H*_3_), 1.27, 1.18 (2 d, *J* = 6.9 Hz for both, minor 2 × *i*-Pr-C*H*_3_), 1.10, 1.01 (2 d, *J* = 6.9 Hz for both, major 2 × *i*-Pr-C*H*_3_); ^13^C-NMR (100 MHz, CDCl_3_) δ (ppm): 166.5, 166.1, 165.5, 165.1 (major 4 × C=O), 166.3, 165.4 (2), 165.2 (minor 4 × C=O), 149.7, 148.3, 148.0 (minor Tria-C-4, Qu-C-2, Qu-C-8a), 149.1, 148.4, 147.9 (major Tria-C-4, Qu-C-2, Qu-C-8a), 141.3 (major Qu-C-4), 140.8 (minor Qu-C-4), 134.1, 133.9, 133.7, 133.6, 133.4, 133.1, 132.5, 130.3-128.6, 128.3, 127.6 (minor and major Tria-C-5, Qu-C-4a, Qu-C-5 – Qu-C-8), 119.3 (major Qu-C-3), 119.1 (minor Qu-C-3), 105.6, 102.4 (major 2 × *p*-cym-C_qAr_), 105.1, 101.7 (minor 2 × *p*-cym-C_qAr_), 88.8 (minor C-1’), 88.7 (major C-1’), 86.0, 85.0, 84.9, 84.4 (minor 4 × *p*-cym-CH_Ar_), 85.2, 84.9, 84.7, 84.5 (major 4 × *p*-cym-CH_Ar_), 73.0, 70.4, 70.3, 67.9 (major C-2’ – C-5’), 72.3, 70.8, 69.2, 68.5 (minor C-2’ – C-5’), 62.1 (minor C-6’), 61.8 (major C-6’), 31.4 (minor *i*-Pr-*C*H), 31.3 (major *i*-Pr-*C*H), 22.9, 21.1 (major 2 × *i*-Pr-*C*H_3_), 22.8, 21.8 (minor 2 × *i*-Pr-*C*H_3_), 18.5 (major C_6_H_4_-*C*H_3_), 18.3 (minor C_6_H_4_-*C*H_3_). ESI-HRMS positive mode (m/z): calculated for C_55_H_48_ClN_4_O_9_Ru^+^ [M-PF_6_]^+^ 1045.2160. Found: 1045.2160.

### Complex Os-4b

Obtained from the chloro-bridged **Os-dimer** (10.0 mg, 0.013 mmol), ligand **4b** (20.6 mg, 0.027 mmol, 2.1 eq.) and TlPF_6_ (8.8 mg, 0.027 mmol) according to general procedure IV. After removal of the precipitated TlCl by filtration and evaporation of the filtrate the residual crude was dissolved in chloroform (2 mL), and diisopropyl ether (8 mL) was added. The precipitation was filtered off, washed with a 1:4 solvent mixture of chloroform-diisopropyl ether (2 × 1 mL) and dried to result in 33 mg of **Os-4b** as an orange powder. Percent yield: 96%; diastereomeric ratio: 5:2. R_f_ = 0.40 (95:5 CHCl_3_-MeOH). ^1^H-NMR (400 MHz, CDCl_3_) δ (ppm): 9.17 (s, major Tria-H-5), 9.04 (s, minor Tria-H-5), 8.70 (d, *J* = 8.8 Hz, minor Qu-H-8), 8.65 (d, *J* = 8.8 Hz, major Qu-H-8), 8.34 (d, *J* = 8.5 Hz, major Qu-H-4), 8.26 (d, *J* = 8.5 Hz, minor Qu-H-4), 8.14 (d, *J* = 8.5 Hz, major Qu-H-3), 8.06 (d, *J* = 8.5 Hz, minor Qu-H-3), 8.10-7.23 (m, minor and major Ph, Qu-H-5 – Qu-H-7), 7.08 (pt, *J* = 9.7, 9.7 Hz, major H-3’), 7.02 (d, *J* = 5.5 Hz, major H-1’), 6.81 (d, *J* = 5.4 Hz, minor H-1’), 6.75 (pt, *J* = 8.3, 8.2 Hz, minor H-3’), 6.36, 6.17 (2), 5.78 (4 d, *J* = 5.6 Hz in each, major 4 × *p*-cym-CH_Ar_), 6.35, 6.20, 6.10, 5.90 (4 d, *J* = 5.6 Hz in each, minor 4 × *p*-cym-CH_Ar_), 6.25 (dd, *J* = 8.7, 5.5 Hz, minor H-2’), 6.05 (pt, *J* = 9.7, 9.6 Hz, major H-4’), 5.98 (dd, *J* = 10.0, 5.5 Hz, major H-2’), 5.89 (pt, *J* = 9.4, 8.1 Hz, minor H-4’), 4.99-4.50 (m, minor and major H-5’, H-6’a,b), 2.73 (hept, *J* = 6.9 Hz, minor *i*-Pr-C*H*), 2.49 (hept, *J* = 6.9 Hz, major *i*-Pr-C*H*), 2.09 (s, major C_6_H_4_-C*H*_3_), 2.02 (s, minor C_6_H_4_-C*H*_3_), 1.14, 1.13 (2 d, *J* = 6.9 Hz for both, minor 2 × *i*-Pr-C*H*_3_), 0.98, 0.96 (2 d, *J* = 6.9 Hz for both, major 2 × *i*-Pr-C*H*_3_); ^13^C-NMR (100 MHz, CDCl_3_) δ (ppm): 166.5, 166.1, 165.4, 165.1 (major 4 × C=O), 166.3, 165.4, 165.3, 165.1 (minor 4 × C=O), 150.3, 149.1, 148.0 (minor Tria-C-4, Qu-C-2, Qu-C-8a), 149.7, 148.9, 148.1 (major Tria-C-4, Qu-C-2, Qu-C-8a), 141.6 (major Qu-C-4), 141.1 (minor Qu-C-4), 134.1, 134.0, 133.9, 133.7, 133.6, 133.4, 133.2, 132.7, 130.4-128.6, 128.3, 127.6 (minor and major Tria-C-5, Qu-C-4a, Qu-C-5 – Qu-C-8), 118.9 (major Qu-C-3), 118.8 (minor Qu-C-3), 96.5, 95.2 (major 2 × *p*-cym-C_qAr_), 95.8, 94.0 (minor 2 × *p*-cym-C_qAr_), 85.1 (minor C-1’), 85.0 (major C-1’), 80.6, 80.5, 75.7, 75.6 (minor 4 × *p*-cym-CH_Ar_), 80.4, 76.6, 76.3, 75.8 (major 4 × *p*-cym-CH_Ar_), 73.1 (major C-5’), 72.5 (minor C-5’), 70.7 (minor C-3’), 70.3 (major C-3’), 70.2 (major C-2’), 69.1 (minor C-4’), 68.4 (minor C-2’), 67.9 (major C-4’), 62.1 (minor C-6’), 61.8 (major C-6’), 31.5 (minor *i*-Pr-*C*H), 31.3 (major *i*-Pr-*C*H), 23.1, 21.3 (major 2 × *i*-Pr-*C*H_3_), 23.0, 22.0 (minor 2 × *i*-Pr-*C*H_3_), 18.5 (major C_6_H_4_-*C*H_3_), 18.4 (minor C_6_H_4_-*C*H_3_). ESI-HRMS positive mode (m/z): calculated for C_55_H_48_ClN_4_O_9_Os^+^ [M-PF_6_]^+^ 1135.2713. Found: 1135.2714.

### Complex Ir-4b

Obtained from the chloro-bridged **Ir-dimer** (10.0 mg, 0.013 mmol), ligand **4b** (19.5 mg, 0.025 mmol, 2.0 eq.) and TlPF_6_ (8.8 mg, 0.025 mmol) according to general procedure IV. After removal of the precipitated TlCl by filtration and evaporation of the filtrate the residual crude was dissolved in chloroform (2 mL), and diisopropyl ether (10 mL) was added. The precipitation was filtered off, washed with a 1:4 solvent mixture of chloroform-diisopropyl ether (2 × 1 mL) and dried to result in 31.4 mg of **Ir-4b** as a yellow powder. Percent yield: 97%; diastereomeric ratio: 3:2. R_f_ = 0.42 (95:5 CHCl_3_-MeOH). ^1^H-NMR (400 MHz, CDCl_3_) δ (ppm): 9.17 (s, major Tria-H-5), 9.04 (s, minor Tria-H-5), 8.47-7.23 (m, minor and major Ph, Qu-H-3 – Qu-H-8), 7.05 (d, *J* = 5.9 Hz, major H-1’), 6.88 (pt, *J* = 8.8, 8.8 Hz, minor H-3’ or H-4’), 6.84 (pt, *J* = 9.8, 9.7 Hz, major H-3’ or H-4’), 6.81 (d, *J* = 5.6 Hz, minor H-1’), 6.22 (dd, *J* = 9.3, 5.5 Hz, minor H-2’), 6.04 (pt, *J* = 9.8, 9.5 Hz, major H-3’ or H-4’), 6.01 (dd, *J* = 10.2, 5.9 Hz, major H-2’), 5.98 (dd, *J* = 9.8, 8.5 Hz, minor H-3’ or H-4’), 4.89-4.46 (minor and major H-5’, H-6’a,b), 1.78 (s, minor Cp*-CH_3_), 1.69 (s, major Cp*-CH_3_); ^13^C-NMR (100 MHz, CDCl_3_) δ (ppm): 166.3, 165.6, 165.1, 165.0 (minor 4 × C=O), 166.1, 166.0, 165.6, 165.0 (major 4 × C=O), 149.9, 149.6, 145.6 (minor Tria-C-4, Qu-C-2, Qu-C-8a), 149.8, 149.1, 145.8 (major Tria-C-4, Qu-C-2, Qu-C-8a), 141.8 (major Qu-C-4), 141.4 (minor Qu-C-4), 134.0, 133.8, 133.7, 133.6, 133.4, 133.3, 132.6, 132.4, 130.4-128.6, 128.0, 127.6 (minor and major Tria-C-5, Qu-C-4a, Qu-C-5 – Qu-C-8), 119.4 (major Qu-C-3), 119.3 (minor Qu-C-3), 90.3 (2) (minor and major Cp*), 85.3 (major C-1’), 85.2 (minor C-1’), 72.7, 70.8, 70.1, 68.1 (major C-2’ – C-5’), 72.5, 70.9, 68.7, 68.3 (minor C-2’ – C-5’), 62.2 (minor C-6’), 61.7 (major C-6’), 9.4 (minor Cp*-*C*H_3_), 9.3 (major Cp*-*C*H_3_). ESI-HRMS positive mode (m/z): calculated for C_55_H_49_ClN_4_O_9_Ir^+^ [M-PF_6_]^+^ 1137.2808. Found: 1137.2808.

### Complex Rh-4b

Obtained from the chloro-bridged **Rh-dimer** (10.0 mg, 0.016 mmol), ligand **4b** (26.3 mg, 0.034 mmol, 2.1 eq.) and TlPF_6_ (11.3 mg, 0.032 mmol) according to general procedure IV. After removal of the precipitated TlCl by filtration and evaporation of the filtrate the residual crude was dissolved in chloroform (2 mL), and diisopropyl ether (8 mL) was added. The precipitation was filtered off, washed with a 1:3 solvent mixture of chloroform-diisopropyl ether (2 × 1 mL) and dried to result in 28 mg of **Rh-4b** as an orange powder. Percent yield: 70%; diastereomeric ratio: 3:2. R_f_ = 0.29 (95:5 CHCl_3_-MeOH). ^1^H-NMR (400 MHz, CDCl_3_) δ (ppm): 9.09 (s, major Tria-H-5), 8.98 (s, minor Tria-H-5), 8.50 (d, *J* = 8.9 Hz, major Qu-H-8), 8.42 (d, *J* = 8.5 Hz, minor Qu-H-8), 8.42 (d, *J* = 8.5 Hz, major Qu-H-4), 8.34 (d, *J* = 8.5 Hz, minor Qu-H-4), 8.07 (d, *J* = 8.5 Hz, major Qu-H-3), 7.99 (d, *J* = 8.5 Hz, minor Qu-H-3), 8.03-7.23 (m, minor and major Ph, Qu-H-5 – Qu-H-7), 7.06 (d, *J* = 5.9 Hz, major H-1’), 6.90 (pt, *J* = 10.0, 9.6 Hz, minor and major H-3’), 6.79 (d, *J* = 5.5 Hz, minor H-1’), 6.20 (dd, *J* = 9.3, 5.5 Hz, minor H-2’), 6.07 (pt, *J* = 9.9, 9.8 Hz, major H-4’), 5.98 (dd, *J* = 9.6, 8.5 Hz, minor H-4’), 5.96 (dd, *J* = 10.3, 5.8 Hz, major H-2’), 4.89-4.45 (m, minor and major H-5’, H-6’a,b), 1.76 (s, minor Cp*-CH_3_), 1.65 (s, major Cp*-CH_3_); ^13^C-NMR (100 MHz, CDCl_3_) δ (ppm): 166.3, 165.5, 165.2, 165.1 (minor 4 × C=O), 166.1, 166.0, 165.7, 165.1 (major 4 × C=O), 148.6, 148.0, 145.9 (minor Tria-C-4, Qu-C-2, Qu-C-8a), 148.1, 147.9, 146.1 (major Tria-C-4, Qu-C-2, Qu-C-8a), 141.3 (major Qu-C-4), 140.9 (minor Qu-C-4), 134.1, 134.0, 133.9, 133.8, 133.6, 133.4, 133.3, 132.2, 132.0, 130.4-128.6, 128.0, 127.5 (minor and major Tria-C-5, Qu-C-4a, Qu-C-5 – Qu-C-8), 119.7 (major Qu-C-3), 119.6 (minor Qu-C-3), 98.2, 98.1, 98.1, 98.0 (minor and major Cp*), 85.0 (major C-1’), 84.9 (minor C-1’), 72.6 (major C-5’), 72.4 (minor C-5’), 70.9 (minor C-3’), 70.7 (major C-3’), 70.5 (major C-2’), 68.7 (minor C-4’), 68.3 (minor C-2’), 68.1 (major C-4’), 62.3 (minor C-6’), 61.7 (major C-6’), 9.6 (minor Cp*-*C*H_3_), 9.5 (major Cp*-*C*H_3_). ESI-HRMS positive mode (m/z): calculated for C_55_H_49_ClN_4_O_9_Rh^+^ [M-PF_6_]^+^ 1047.2238. Found: 1047.2237.

### Complex Os-5a

Obtained from the chloro-bridged **Os-dimer** (8.0 mg, 0.010 mmol), ligand **5a** (13.7 mg, 0.021 mmol, 2.1 eq.) and TlPF_6_ (7.1 mg, 0.020 mmol) according to general procedure IV. After removal of the precipitated TlCl by filtration and evaporation of the filtrate the residual crude was purified by column chromatography (100:5 CHCl_3_-MeOH) to result in 22.5 mg of **Os-5a** as a yellow solid. Percent yield: 90%; diastereomeric ratio: 1:1. R_f_ = 0.33 (95:5 CHCl_3_-MeOH). ^1^H-NMR (400 MHz, CDCl_3_) δ (ppm): 9.20, 9.14 (2 × 1H, 2 d, *J* = 5.7 Hz for both, 2 × Py-H-6), 8.84, 8.71 (2 × 1H, 2 s, 2 × Tria-H-5), 8.10-7.91 (4H, m, 2 × Py-H-3, 2 × Py-H-4), 7.50, 7.45 (2 × 1H, ddd, *J* = 7.4, 5.7, 1.3 Hz for both, 2 × Py-H-5), 6.54, 6.45, 6.29 (2), 6.16, 6.10, 6.04, 5.95, 5.90, 5.85 (2 × 5H, 10 d, 2 × H-1’, 2 × 4 × *p*-cym-CH_Ar_), 6.51, 6.26 (2 × 1H, 2 pt, *J* = 10.1, 10.1 Hz and 9.5, 9.4 Hz, respectively, 2 × H-3’ or H-4’), 5.65, 5.51 (2 × 1H, 2 dd, *J* = 10.4, 5.7 Hz and 9.7, 5.6 Hz, respectively, 2 × H-2’), 5.40, 5.27 (2 × 1H, 2 pt, *J* = 9.9, 9.9 Hz and 9.6, 9.5 Hz, respectively, 2 × H-3’ or H-4’), 4.35-4.05 (6H, m, 2 × H-5’, 2 × H-6’a,b), 2.86, 2.73 (2 × 1H, 2 hept, *J* = 6.9 Hz for both, 2 × *i*-Pr-C*H*), 2.41-0.74 (90H, m, 2 × 2 × *i*-Pr-C*H*_3_, 2 × C_6_H_4_-C*H*_3_, 2 × 4 × C*H*_3_C*H*_2_C*H*_2_C*H*_2_CO); ^13^C-NMR (100 MHz, CDCl_3_) δ (ppm): 173.4, 173.3, 173.2, 172.7, 172.6, 172.4, 172.2, 172.0 (2 × 4 × C=O), 155.4, 154.8 (2 × Py-C-6), 149.0, 148.5, 148.4, 147.8 (2 × Tria-C-4, 2 × Py-C-2), 140.5, 140.2 (2 × Py-C-4), 129.9, 129.6 (2 × Tria-C-5), 127.6, 127.1 (2 × Py-C-3), 123.3, 122.9 (2 × Py-C-5), 96.6, 95.2, 94.9, 92.8 (2 × 2 × *p*-cym-C_qAr_), 85.0, 84.5 (2 × C-1’), 81.1, 79.3, 76.8, 76.7, 75.6, 75.4, 74.9, 73.9, 72.7, 71.7, 70.1, 69.4, 68.6, 68.2, 68.0, 67.1 (2 × 4 × *p*-cym-CH_Ar_, 2 × C-2’ ‒ C-5’), 61.0, 60.8 (2 × C-6’), 33.9-33.1 (2 × 4 × *C*H_2_), 31.2, 31.1 (2 × *i*-Pr-*C*H), 27.0-26.6 (2 × 4 × *C*H_2_), 23.5, 22.7, 22.0, 21.4 (2 × 2 × *i*-Pr-*C*H_3_), 22.4-22.1 (2 × 4 × CH_2_), 18.3, 18.0 (2 × C_6_H_4_-*C*H_3_), 13.8-13.7 (2 × 4 × *C*H_3_). ESI-HRMS positive mode (m/z): calculated for C_43_H_62_ClN_4_O_9_Os^+^ [M-PF_6_]^+^ 1005.3807. Found: 1005.3808.

# Copies of the ^1^H and ^13^C NMR spectra

**2a** (CDCl_3_, 400 MHz)

**2a** (CDCl_3_, 100 MHz)

**2b** (CDCl_3_, 400 MHz)

**2b** (CDCl_3_, 100 MHz)

**3a** (CD_3_OD, 400 MHz)

**3a** (CD_3_OD, 100 MHz)

**3b** (CD_3_OD, 400 MHz)

**3b** (CD_3_OD, 100 MHz)

**4a** (CDCl_3_, 400 MHz)

**4a** (CDCl_3_, 100 MHz)

**4b** (CDCl_3_, 360 MHz)

**4b** (CDCl_3_, 90 MHz)

**5a** (CDCl_3_, 360 MHz)

**5a** (CDCl_3_, 90 MHz)

**Ru-2a** (CDCl_3_, 400 MHz)

**Ru-2a** (CDCl_3_, 100 MHz)

**Ir-2a** (CDCl_3_, 400 MHz)

**Ir-2a** (CDCl_3_, 100 MHz)

**Ru-2b** (CDCl_3_, 400 MHz)

**Ru-2b** (CDCl_3_, 100 MHz)

**Ir-2b** (CDCl_3_, 400 MHz)

**Ir-2b** (CDCl_3_, 100 MHz)

**Ru-3a** (CD_3_OD, 400 MHz)

**Ru-3a** (CD_3_OD, 100 MHz)

**Ir-3a** (CD_3_OD, 400 MHz)

**Ir-3a** (CD_3_OD, 100 MHz)

**Ru-4a** (CDCl_3_, 400 MHz)

**Ru-4a** (CDCl_3_, 100 MHz)

**Os-4a** (CDCl_3_, 400 MHz)

**Os-4a** (CDCl_3_, 100 MHz)

**Ir-4a** (CDCl_3_, 400 MHz)

**Ir-4a** (CDCl_3_, 100 MHz)

**Rh-4a** (CDCl_3_, 400 MHz)

**Rh-4a** (CDCl_3_, 100 MHz)

**Ru-4b** (CDCl_3_, 400 MHz)

**Ru-4b** (CDCl_3_, 100 MHz)

**Os-4b** (CDCl_3_, 400 MHz)

**Os-4b** (CDCl_3_, 100 MHz)

**Ir-4b** (CDCl_3_, 400 MHz)

**Ir-4b** (CDCl_3_, 100 MHz)

**Rh-4b** (CDCl_3_, 400 MHz)

**Rh-4b** (CDCl_3_, 100 MHz)

**Os-5a** (CDCl_3_, 400 MHz)

**Os-5a** (CDCl_3_, 100 MHz)

# References

Godó, A.J., Bényei, A.C., Duff, B., Egan, D.A., and Buglyó, P. (2012). Synthesis and X-ray diffraction structures of novel half-sandwich Os(II)-and Ru(II)-hydroxamate complexes. *RSC Adv.* 2**,** 1486-1495. doi: 10.1039/C1RA00998B

Son, M.H., Kim, J.Y., Lim, E.J., Baek, D.J., Choi, K., Lee, J.K., et al. (2013). Synthesis and biological evaluation of 2-(arylethynyl)quinoline derivatives as mGluR5 antagonists for the treatment of neuropathic pain. *Bioorg. Med. Chem. Lett.* 23**,** 1472-1476. doi: 10.1016/j.bmcl.2012.12.056

Zhang, H., Wang, Y., Thürmer, R., Parvez, K., Choudhary, I., Rahman, A.-u., et al. (1999). Neighbouring Group Participation of C-6 Substituents of Glucose Derivatives on the Stereoselectivity of the *N*-Glycosidic Linkage of Glycopeptides. *Z. Naturforsch. B* 54**,** 692-698. doi:10.1515/znb-1999-0519
